# Darunavir-Resistant HIV-1 Protease Constructs Uphold a Conformational Selection Hypothesis for Drug Resistance

**DOI:** 10.3390/v12111275

**Published:** 2020-11-08

**Authors:** Zhanglong Liu, Trang T. Tran, Linh Pham, Lingna Hu, Kyle Bentz, Daniel A. Savin, Gail E. Fanucci

**Affiliations:** 1 Department of Chemistry, University of Florida, Gainesville, FL 32611, USA; zhanglong.liu@gmail.com (Z.L.); trangtran@ufl.edu (T.T.T.); linhpham@tamuct.edu (L.P.); lnhu39@chem.ufl.edu (L.H.); kcbentz@chem.ufl.edu (K.B.); savin@chem.ufl.edu (D.A.S.); 2 LinkedIn, Mountain View, Sunnyvale, CA 94043, USA; 3 Department of Science and Mathematics, Texas A&M University—Central Texas, Killeen, TX 76549, USA; 4 Department of Chemistry and Biochemistry, University of California, San Diego, La Jolla, CA 92093, USA

**Keywords:** HIV-1 protease, darunavir, genetic and phenotypic diversity, DEER spectroscopy, drug resistance

## Abstract

Multidrug resistance continues to be a barrier to the effectiveness of highly active antiretroviral therapy in the treatment of human immunodeficiency virus 1 (HIV-1) infection. Darunavir (DRV) is a highly potent protease inhibitor (PI) that is oftentimes effective when drug resistance has emerged against first-generation inhibitors. Resistance to darunavir does evolve and requires 10–20 amino acid substitutions. The conformational landscapes of six highly characterized HIV-1 protease (PR) constructs that harbor up to 19 DRV-associated mutations were characterized by distance measurements with pulsed electron double resonance (PELDOR) paramagnetic resonance spectroscopy, namely double electron–electron resonance (DEER). The results show that the accumulated substitutions alter the conformational landscape compared to PI-naïve protease where the semi-open conformation is destabilized as the dominant population with open-like states becoming prevalent in many cases. A linear correlation is found between values of the DRV inhibition parameter K_i_ and the open-like to closed-state population ratio determined from DEER. The nearly 50% decrease in occupancy of the semi-open conformation is associated with reduced enzymatic activity, characterized previously in the literature.

## 1. Introduction

Human immunodeficiency virus 1 (HIV-1) protease (PR) is a potent target in the treatment of HIV-1 infection because its inhibition leads to non-infectious immature virus particles [1,2,3,4,5]. Protease inhibitors (PIs) in combination with other classes of anti-HIV drugs given in antiretroviral therapies (ARTs) are very successful in keeping viral loads below detectable limits within the blood. However, the emergence of multidrug resistance is a roadblock to the successful suppression of undetectable viral loads in infected patients, and as such, there is great interest in understanding the mechanisms of drug resistance [6,7,8].

**Figure 1:**
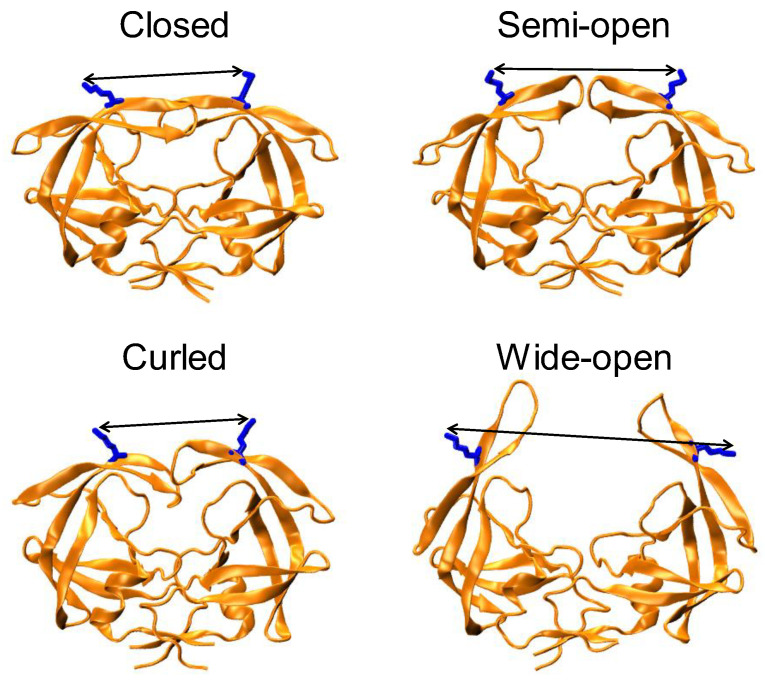
Four representative conformations of populations to describe HIV-1 PR conformational landscape, namely closed ~ 33 Å (PDBID: 2BPX), semi-open ~ 36 Å (PDBID: 1HHP), curled ~ 25-30 Å (MD coordinates) and wide-open > 40 Å (MD coordinates). Residues K55 and K55′ are rendered in blue stick format with the side chain distance between them shown as arrows.

Our lab has utilized distance measurements from double electron–electron resonance (DEER) spectroscopy [9,10,11], to formulate a conformational landscape hypothesis that describes how amino acid substitutions combine to impact drug resistance and restore kinetic fitness in HIV-1 PR. In our model, we postulate that drug-pressure selected mutations combine to stabilize open-like states (either wide-open or curled/tucked) and destabilize closed-like conformations [12,13,14,15,16,17,18,19]. This conformational sampling scheme encompasses four conformational ensembles described as curled/tucked, wide-open, semi-open, and closed (Figure 1). These conformations are proposed from a combination of X-ray structures, molecular dynamic (MD) simulations, and our DEER data [19,20]. Our prior work also shows that as the fractional occupancy of the open-like conformations become more highly populated, there is an overall concomitant increase in protein backbone dynamics determined from nuclear magnetic resonance (NMR) spectroscopy [16,19]. This conformational selection hypothesis can be operating in addition to drug resistance produced by other mechanisms including structural alterations to the binding site cavity, distal mutations that alter dimerization/subunit interactions, gag/pol substrate processing, and protease dynamics [21,22,23,24,25,26,27,28,29].

One of our earlier studies focused on the specific accumulation of amino acid changes in response to nelfinavir (NFV) treatment, specifically the D30N primary mutation with the accumulation of secondary mutations M36I and A71V [14,30]. We also investigated the impact of accumulated mutations in three clinical isolate sequences that demonstrated multidrug resistance [12,13,15,18]. Here, we extend the investigation to a set of darunavir (DRV)-resistant sequences that were generated via analysis of mutated clinical derived sequences from subtype B [31]. Darunavir is the most recently approved HIV-1 PR inhibitor, and it shows a high genetic barrier to resistance [32]. However, resistance has been clinically reported and understanding mechanisms for resistance is important for the early detection of treatment failure and design of next generation PIs capable of inhibiting multidrug-resistant virus [28,33,34,35,36,37,38,39,40,41,42].

**Figure 2:**
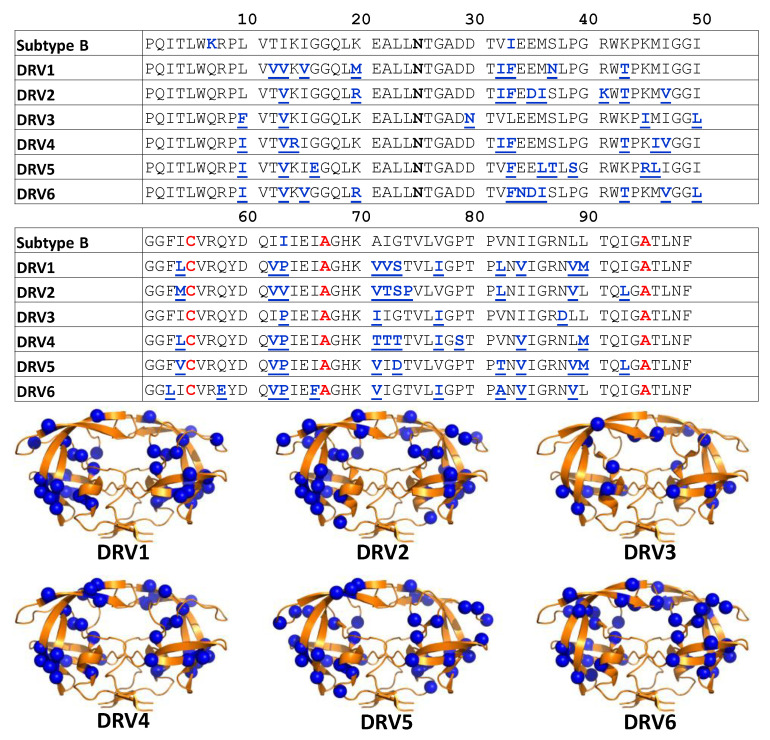
(**Top**) Graphical table showing darunavir (DRV) 1-6 HIV-1 PR sequences with boldfaced underlined residues in blue indicating substitutions relative to PI-naïve subtype B. Blue bold-face residues in sequence of PI-naïve subtype B indicate stabilizing mutations (Q7K, L33I, and L63I). Black boldfaced annotation for D25N shows these constructs contained an inactivation of the catalytic site to aid in stabilization. Red boldfaced labels indicate locations modified for DEER investigations as described in the Materials and Methods. (**Bottom**). Ribbon diagrams of HIV-1PR (PDBID: 2PK5) with spheres showing the locations of the amino acid substitutions in DRV1-6 relative to PI-naïve subtype B.

The sequences of HIV-1 PR targeted for this study are given in Figure 2 with the location of the amino acid changes shown as spheres in ribbon diagrams. Kinetic and inhibition parameters have been previously characterized for these constructs [31]; structural information also exists for these or other DRV-resistant constructs [43,44,45]. Thus, they readily provide a set of constructs to add to our postulated model of conformational selection for understanding multidrug resistance and enzymatic activity. DRV-resistance oftentimes results in >18 amino acid changes, and these constructs represent the most highly mutated PR sequences we have investigated by DEER spectroscopy to date. Our earlier work on three multidrug-resistant constructs had 10, 7, and 10 mutations, respectively for constructs termed POST [12], V6 [15,18], and MDR769 [13].

Overall, the results for these DRV-resistant constructs uphold a conformational landscape model where a correlation between the ratio of the open-like to closed-like states to inhibition values is observed. This trend indicates a flip-flop in the stability of the open-like to closed-like states, with drug-pressure selected mutations stabilizing open-like states. This seems reasonable given that current inhibitors are modeled after the transition-state analog of the substrate, which binds and induced a closed conformation of the enzyme. Results further suggest a possible on–off switch for kinetic turn-over that requires the semi-open population of the unliganded enzyme to predominate (>60% relative population) for efficient activity. Together, these findings suggest the consideration of open-like conformations, and non-active-site inhibitor binding, as potential targets for novel inhibitor design strategies.

## 2. Materials and Methods

### 2.1. Cloning and Mutagenesis

DNA, which was mRNA stabilized and codon optimized for expression in *Escherichia coli*, that encodes for each of the DRV sequences given in Figure 2 was purchased from DNA 2.0 (Meno Park, CA, USA). Genes were subcloned into pET-23a vectors (Novagen, Madison WI, USA) under the control of the T7 promoter. DRV constructs included three stabilizing mutations, Q7K, L331, and L63I, which we have typically included in our DEER investigations of HIV-1 PR, as we desired to match our protein samples as closely to those previously studied [20,46]. These sites are omitted if one of these locations is a natural polymorphism or drug-pressure selected mutation. HIV-1 PR is a homodimer, so one CYS substitution generates a pair of spin labels for distance measurements. For spin-labeling, a unique cysteine at site 55 is incorporated, which has been shown not to alter enzyme activity [47,48], and which we have shown can be readily spin-labeled as well as tolerate a fluorescent tag without protein precipitation/aggregation [49]. We initially chose site K55C based upon analysis of all HIV-1 PR structures in the Protein Data Bank in 2005 analyzing distance between terminal lysine amine groups that predicted ≈ 3 Å difference should be observed in our DEER data between the inhibitor-bound closed conformation and the unbound semi-open states. We have demonstrated that this single spin-labeled site reports changes in distances and distance distributions between the major conformations detected in numerous X-ray structures of closed (33 Å) and open (36 Å), and we find additional distance populations reflective of two other conformational states described as wide-open (>40 Å) and curled/tucked (25–30 Å) [16,50,51]; these results have been substantiated by MD simulations [15,52] and crystallographic investigations [16,51]. To ensure unique labeling, the two naturally occurring cysteine residues are substituted with (C67A, C95A), which is often done in crystallographic studies to prevent disulfide bond formation and limit protein aggregation [47,53]. To facilitate spectroscopic studies, all samples for DEER spectroscopy contain the D25N mutation, and we have shown that this mutation does not impact the trends of inhibitor binding [16,17]. The fidelity of the HIV-1 PR gene sequence was confirmed by Sanger DNA sequencing (ICBR Genomics Facility, University of Florida).

### 2.2. Protein Expression, Purification, and Spin-Labeling

Protein was expressed as described in previous publications, with adjustment of the pH of the inclusion body buffer for anion exchange [12]. We find that the isoelectric point of HIV-1PR is altered upon amino acid substitution, and we alter purification buffer pH to optimize purification conditions that prevent protein aggregation. Buffers were adjusted to pH 7.14, 8.52, 8.52, 8.55, 7.14, and 8.55; for DRV1, DRV2, DRV 3, DRV4, DRV5, and DRV6; respectively. Protein was spin labeled with MTSL (1-Oxyl-2,2,5,5-Tetramethyl-Δ3-Pyrroline-3-Methyl) Methanethiosulfonate (Santa Cruz Biotechnology, CA, USA), freshly dissolved in ethanol, in a 5–10× excess of the protein concentration. The reaction was carried out in 10 mM Tris-HCl buffer pH 6.9 for 6–12 h in the dark at 4 °C because protease is found to precipitate if the labeling is performed at room temperature. After the reaction, excess spin-label was removed by buffer exchange into 2 mM NaOAc pH 5.0 using HiPrep 26/10 desalting columns. Spin-labeling was confirmed through mass spectrometry analysis (Table S1 and Figure S1). Accurate mass experiments were performed on an Agilent 6220 ESI TOF (Santa Clara, CA, USA) mass spectrometer equipped with an electrospray source operated in positive ion mode. Agilent ESI Low Concentration Tuning Mix was used for mass calibration for a calibration range of *m*/*z* 100–2000. Samples were prepared in a solution containing acidified acetonitrile (0.5% formic acid), and 1 μL was injected into the electrospray source at a rate of 100 mL min^−1^. Optimal conditions were capillary voltage 4000 V, source temperature 350 °C, and a cone voltage of 60 V. The time-of-flight analyzer was scanned over an appropriate *m*/*z* range with a 1 s integration time. Data were acquired in continuum mode until the acceptable averaged data was obtained. ESI results were collected for all samples, and complete spin labeling of proteins was confirmed with correctly anticipated masses before proceeding to DEER data collection.

### 2.3. Sample Preparation, DEER Data Collection, and Analysis

For DEER spectroscopy, samples were further concentrated and buffer exchanged to 100–140 μM dimer concentration in 20 mM D_3_-NaOAc/D_2_O, pH 5.0 with 30% *v/v* D_8_-glycerol by buffer exchange using centrifugal membrane concentrators (Millipore, Billerica, MA, USA). For DRV1 and DRV3 unbound HIV-1 PR, aggregation problems were encountered in the sodium acetate buffer at pH 5, as evidenced by continuous wave (CW) X-band electron paramagnetic resonance (EPR) line shapes and dynamic light scattering (DLS) (Figures S2–S4) [54]. Various pH conditions were explored, and homogenous protein samples with the high concentration of around 100 μM were only obtained at pH 2.8–3.0 (Figures S2–S4). Our lab has performed solution NMR and X-ray crystallography of HIV-1 PR in the past, so we have experience in knowing what spectroscopic signatures in CW EPR line shapes signify homogeneous samples, and the supporting DLS data help verify sample integrity [12,17,19]. Samples with inhibitors were prepared by adding a 4-fold molar excess of inhibitor followed by equilibration at room temperature for four hours prior to freezing in liquid nitrogen for DEER measurements. The ratio of inhibitor:PR was determined from earlier NMR titration experiments [17,19]. Inhibitors were obtained from the NIH AIDS Research and Reference Reagent Program, Division of AIDS, NIAID, NIH, and the non-hydrolysable CaP2 substrate mimic (H-Arg-Val-Leu-r-Phe-Glu-Ala-Nle/NH2 (r = reduced)) was purchased from Peptides International (Louisville, KY, USA). CW EPR spectra were collected at room temperature on a Bruker E500 spectrometer with a Bruker dielectric resonator. Spectra were reported as an average of 16 scans with 100 G sweep width, 0.8 G modulation amplitude, 100 kHz modulation frequency, and 2 mW incident microwave power. CW spectra serve as a control for sample quality prior and after DEER experiments. All DEER experiments were performed on a Bruker EleXsys E580 spectrometer at 65 K with an ER 4118X-MD5 dielectric split-ring resonator. Samples were flash frozen in liquid nitrogen before being inserted into the resonator. The four-pulse DEER sequence was utilized as described previously [14,16,19,55]. Distance profiles are determined by Tikhonov regularization (TKR) as implemented within DEERAnalysis2019 (http://www.epr.ethz.ch/software.html) [9,10,11]. Population analysis proceeds via Gaussian reconstruction and peak suppression of the DEER distance profile as outlined previously [14,19,55,56,57]. The complete details of data analysis are provided in the Supporting Information (Figures S5–S24).

## 3. Results

### 3.1. DRV-Resistant Constructs Sample High Fractional Occupancy of Open-Like and Closed State Compared to PI-Naïve Subtype B

**Figure 3:**
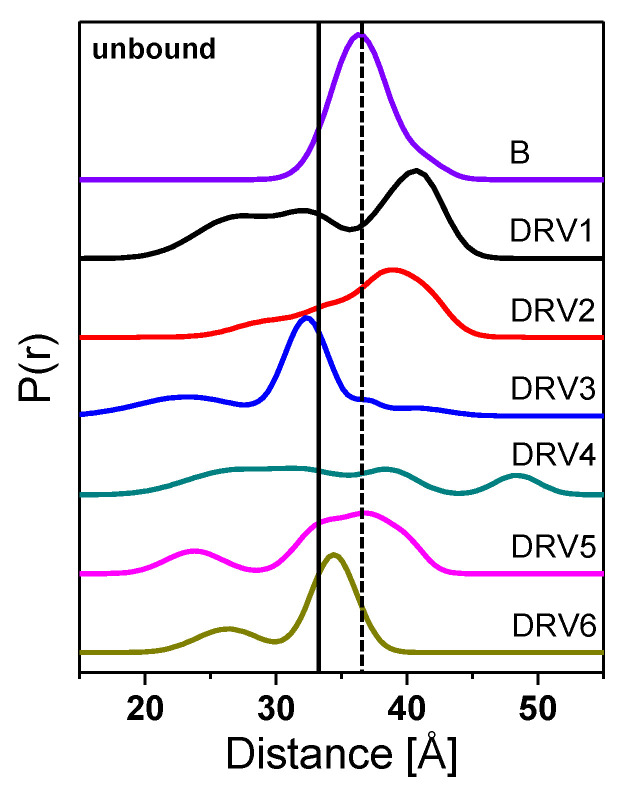
Double electron-electron resonance (DEER) distance probability profiles for unbound HIV-1 PR PI-naïve subtype B, DRV1, DRV2, DRV3, DRV4, DRV5, and DRV6, from top to the bottom. Profiles are area normalized to 100% probability distribution, P(r), and are vertically offset for clarity. Dashed line at 36 Å represents the purported distance observed for HIV-1PR semi-open population, whereas the solid line at 33 Å signifies the distance observed for the HIV-1PR closed population.

Because HIV-1 PR is a homodimer, the incorporation of a single spin label into the protein at site K55C provides a spin-pair for distance measurements by DEER [15,20,47]. Figure 3 shows DEER distance profiles of spin-labeled HIV-1 PR DRV-resistant constructs compared to PI-naïve subtype B (details of data processing of DEER echo curves to generate final distance profiles is provided in Supporting Information). The data clearly reveal marked alterations in the conformational sampling landscape of these DRV-resistant constructs relative to PI-naïve subtype B, particularly with a greater probability of sampled distances < 30 Å, which we assign to a curled/tucked conformation [14,16,19], and distances > 40 Å, corresponding to a wide-open conformation [12,16,18,19,20,55,57].

Table 1 summarizes the most probable distances and the average distances obtained from DEER distance profiles in Figure 3. For unbound HIV-1PR, DRV 5 and DRV6 have most probable distances most similar to PI-naïve subtype B, whereas DRV1 and DRV2 have the most probable distances markedly longer than that seen in PI-naïve subtype B, with DRV3 and DRV4 having shorter ones.

**Figure 4:**
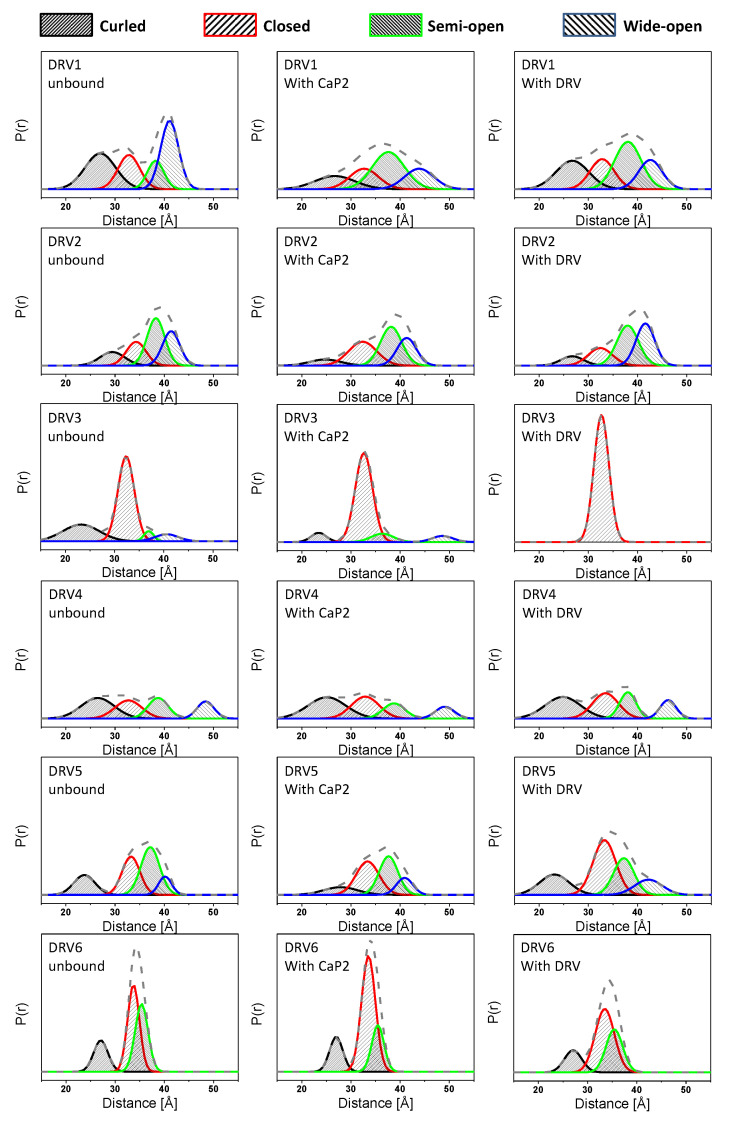
Population analysis of normalized DEER distance profiles for each construct unbound, upon addition of non-hydrolysable CaP2 inhibitor or DRV inhibitor. The gray dashed line represents the overall population profile shown in Figure 3. The curled open conformation is rendered in black with tight forward hashes. The closed population is drawn with red with moderate spaced forward hashes. The semi-open conformation is represented by a green line with tight back hash lines, and the wide-open conformation is in blue with moderately spaced back hash lines. Given the signal-to-noise ratio for collected DEER echo traces, the error for populations is ±3% P(r). Full details of data processing are given in the supporting information and follow the protocol described previously [55,56].

**Figure 5:**
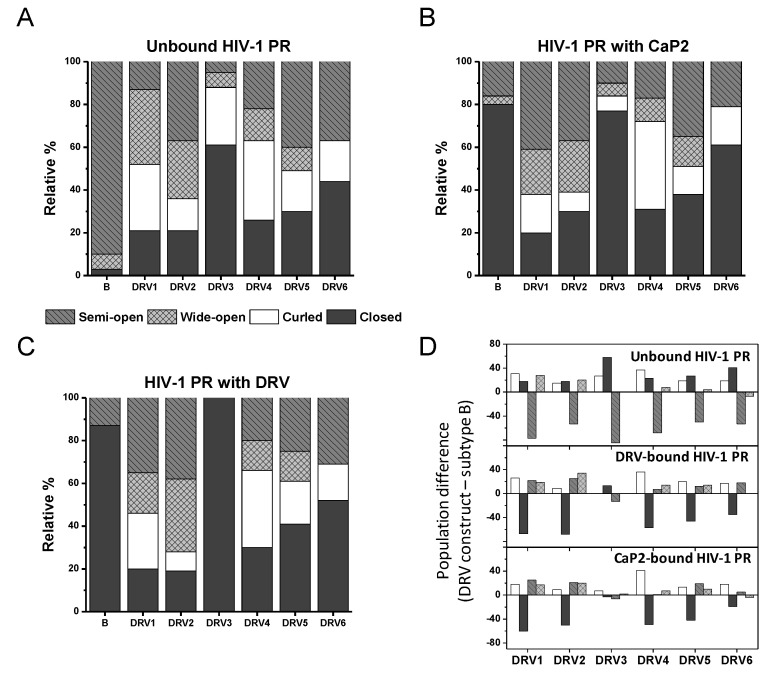
Graphical representation of the relative populations of each of the four conformational states for each HIV-1 PR construct in (**A**) an unbound state, (**B**) addition of the non-hydrolysable substrate analog Ca-P2, and (**C**) addition of inhibitor DRV. (**D**) plots the difference in the population of each state for each DRV construct relative to PI-naïve subtype B.

Although comparing the most probable distances reveals a trend in an average change in the conformational landscape, the DEER distance profile can be modeled to generate a fractional occupancy, *f*_i_, of each of the four HIV-1 PR conformations shown in Figure1, termed curled/tucked- open, closed, semi-open, and wide-open where spin-labels at site K55C generate populations nominally centered at 25–30, 33, 36, and 40–45 Å; respectively [19,55,56]. DEER distance profiles are hence reconstructed as a series of Gaussian-shaped populations representative of the conformational landscape comprising four ensembles [55]. Population analysis of DEER distance profiles for all six DRV constructs in the absence of inhibitor (unbound form) and in the presence of inhibitors Ca-P2 (a non-hydrolysable substrate) and DRV are shown in Figure 4, with full details of the data analysis presented in the Supporting Information in Figures S5–S23. Table 2 summarizes the relative percentages of each conformation, with Table S2 providing values of the population means, breadths, and errors. Figure 5 and Figure S24 plot these values graphically, clearly showing that each DRV construct has a conformational sampling profile that differs markedly from PI-naïve subtype B. By graphing the difference in each population of the DRV constructs relative to PI-naïve subtype B (Figure 5D), we can conclude that in the absence of inhibitor, each DRV construct relative to PI-naïve subtype B has less population of the semi-open state (*p* = 0.001), with in all cases a concomitant increase of the closed (*p* = 0.01), and open-like states, where open-like is the sum of the curled/tucked-open and wide-open populations (*p* = 0.01, save DRV6 *p* = 0.185).

In the unbound form, all DRV constructs sample higher relative percentages of the open-like states (curled/tucked and wide-open) than PI-naïve subtype B. DRV1 and DRV2 occupy roughly 35 ± 5% and 27 ± 5% of a wide-open ensemble; respectively, and DRV5 and DRV6 each sample 19 ± 5% of a curled/tucked conformation. Whereas for unbound DRV3 and DRV4, curled/tucked conformations become the most populated states with fractional occupancies of 27 ± 5% and 36 ± 5%, respectively. Together, the DEER data for these DRV constructs contain populations of these open-like conformations at statistically significantly higher percentages what we observe for subtype B (7 ± 4% and 4 ± 4% for wide-open and curled-tucked; respectively) [14,20], see Supplementary Information Tables S3–S6 for z-test analysis of data. In addition, the breadth of the curled-tucked populations are quite broad for many constructs (8–11 Å, Table S2), possibly reflecting great heterogeneity in flap conformation or possibly even an instability of the dimer; although we did not pursue any thermal stability investigations, we infer this through the pH sensitivity of DRV1 and DRV3. Interestingly, DRV3 and DRV6 have a relatively high population of a closed-like state (61 ± 5%) centered near 33 Å. We have observed several other constructs containing single point or multiple amino acid substitutions, such as natural polymorphisms (NPs) or secondary mutations, that induce a conformation that strongly reflects the closed state [12,14], and for the single point mutant A73V or L63P, we crystallized this protein in the absence of inhibitor and obtained a structure (PDB ID: 5T84) strongly resembling the inhibitor-closed form of the protease.

### 3.2. The Conformational Landscape of Most DRV-Resistant Constructs Is Not Altered by the Addition of DRV or Substrate Mimic

Table 1 and Table 2 also show the analysis of DEER results for DRV-resistant constructs in the presence of a non-hydrolysable substrate analog CaP2 or DRV. In most cases, except for DRV3, very little to no change in the distance probability profile is observed upon the addition of these ligands. This effect can be seen in Figure 4 by comparing the DEER distance distribution profiles (that also contain the population analysis results) from left (unbound state) to right in the middle panel (with CaP2) and the right panel (with DRV). In most cases, minor to no changes can be observed. However, for DRV3, the addition of CaP2 and DRV alter the conformational landscape by removing population density of the non-closed states, which is similar to the behavior of PI-naïve subtype B according to our previous studies [20].

Figure 5A–C plot the relative population (i.e., the fractional occupancy) of each of the four states for each DRV construct in the absence and presence of inhibitor (either Ca-P2 or DRV). Figure 5D plots the relative difference in each fractional occupancy relative to PI-naïve subtype B for DRV constructs, showing that there is a marked decrease in the inhibitor-induced closed population relative to the PI-naïve subtype B for all DRV constructs except for DRV3. These results are in stark contrast to many of our earlier studies where the CaP2 substrate analog usually bound to HIV-1 PR and shifted the conformational ensemble to typically 98% or greater fractional occupancy of the closed state [13,17,19,20]. Given the fold change in K_m_ values reported for these constructs (ranging from ≈1–9× wild-type (WT) values) [31], it may not be surprising that we observed little to no conformational shift with CaP2. We do note that our constructs have the D25N mutation, which may enhance this observed effect, as it is known that the hydrogen bonding interaction of inhibitors with the active site add stabilization energy that is mitigated when the aspartic acid is replaced with an asparagine [16,17], which has been shown to lower binding affinities by 100–1000 fold [58]. Nevertheless, in our earlier studies, except for when we characterized a construct that had a co-evolved substrate [12], CaP2 induced a strong shift to the closed state even with the D25N substitution. DRV3 showed the most dramatic alterations in the conformational landscape upon the addition of CaP2 and DRV, where the addition of these inhibitors removed the non-closed populations, similar to our earlier studies [14,16,17,19,20]. This finding for DRV3 can be understood given that published kinetic and inhibition studies report that the K_m_, k_cat_/K_m_, and K_i_ values for inhibitors Lopinavir (LPV) and DRV) are most similar to PI-naïve subtype B compared to the other DRV analogs [31]. For other DRV constructs, little to no change in the DEER distance profile was observed upon the addition of DRV, which is consistent with K_i_ values that ranged from ≈32 to 2000× WT values [31].

### 3.3. Conformational Landscape Hypothesis for Catalytic Turnover Is Upheld

**Figure 6:**
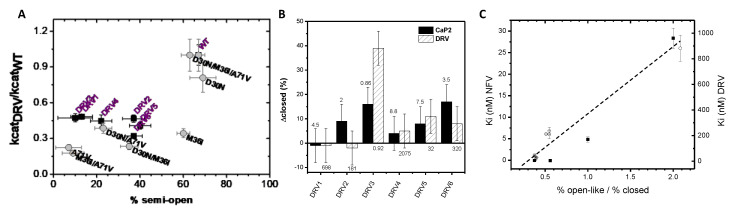
(**A**) Ratio of kcat_DRV_/kcat_WT_ for each construct versus the fractional occupancy of the semi-open conformation in unbound HIV-1 PR. Data are included for the six DRV constructs (black squares) as well as for a series of accumulated mutations in response to nelfinavir (NLV) (gray circles)–taken from [14]. (**B**) Plot of the change in the closed population (∆closed% = closed (inhibitor)–closed (unbound)) for each DRV construct with solid bars being the non-hydrolysable CaP2 inhibitor and slashed bars data for the DRV inhibitor. Numbers above the bar report the fold change in *K*_m_ values, whereas numbers below the bars report the fold change in *K*_i_ values (data taken from [31]). (**C**) Plot of *K*_i_ value as a function of the fractional occupancy of the ratio of the open-like to closed states for unbound DRV constructs with *K*_i_(DRV) plotted as solid squares compared to our earlier investigations of NFV-resistant constructs to *K*_i_(NLV) plotted as open circles. The dashed line is a guide for the eyes showing a linear trend. The y-axes differ for the two data sets and are labeled according to the inhibitor.

The conformational flexibility of HIV-1 PR is well known to be essential for kinetic activity [26]. Results from earlier DEER investigations on nelfinavir (NFV)-resistant constructs suggested that the semi-open conformation is essential for catalytic turnover [14]. Figure 6A plots the relative ratio of the catalytic rate (k_cat (DRV)_/k_cat (WT)_) for each construct as a function of the percentage of the semi-open conformation for the six DRV constructs. This figure also contains data obtained for the accumulated D30N/M36I/A71V NFV resistance mutations [14]. All of the DRV constructs have conformational landscapes that occupy < 50% of the semi-open conformation, which is significantly less than that seen in PI-naïve subtype B (Figure 5A and top panel of Figure 5D) and corresponds with catalytic turnover that is less than half that of the wild-type enzyme (Figure 6A). Numerous studies of DRV-resistant constructs have reported consistent findings with enzymatic activity less than WT [34,36,38]. The DEER population analysis reported here upholds a concept that enzymatic efficiency is obtained by a predominant (>60%) semi-open conformation of protease, where the drug resistance mutations combine to alter conformational sampling that corresponds well to the predicted correlation with kinetic activity [14].

Figure 6B plots for each DRV construct the change in the fractional occupancy of the closed conformation, Δclosed, upon the addition of CaP2 or DRV. Numbers on top of the bars reflect the fold change in K_m_, and those reported below the bars reflect the fold change in K_i_. As expected, DRV3 has a marked conformational shift in the presence of the inhibitor DRV. Other constructs have trends in the shift to the closed state that parallel kinetic and inhibition parameters, meaning as the fold change increases, less of a conformational shift is observed. These results are also seen in the data in Figure 5D for CaP2 and DRV bound showing that less of the closed population is observed compared to PI-naïve subtype B. Although we observed this relationship between the conformational shift and the fold change, the change in closed population is far from a quantitative characterization of the fold change of K_i_.

Figure 6C plots K_i_ values for DRV for the DRV constructs investigated here versus the ratio of the open-like population to closed state, as we had done previously for a series of NFV-accumulated mutations in subtype B [14]. We again find a correlation with the increase in the K_i_ values to the stabilization of open-like states (open like = wide open + curled/tucked open) relative to the stability of the closed state. However, we should note that the relative changes in Ki values have dramatically distinct and independent slopes (≈15 nM for NFV-resistant PR vs. ≈500 nM for DRV-resistant PR). The current result together with our previous finding suggested the ratio of open-like to closed population as an alternative and uniform way to evaluate how conformational sampling can impact HIV-1 PR drug resistance

## 4. Discussion

There have been continued efforts to understand how mutations that accumulate distal from the active site in HIV-1 PR, and in other viral or cancer related proteins, alter enzymatic activity and impart resistance. For HIV-1 PR, others have indicated that some secondary mutations (i.e., drug-pressure selected mutations that are not within the active site cavity) alter the manner in which the extended substrate interacts with PR, which is perhaps important in initial protease cleavage events [39]. It is also possible that distal mutations can impact dimerization or interactions with other HIV-1 or host proteins, including altering protease dynamics [24,25,27,28,29]. We have utilized both DEER and NMR spectroscopies to characterize how the accumulation of secondary drug-pressure selected mutations (which are also natural polymorphisms in other HIV-1PR clades) alter the conformational landscape and protein dynamics. The model emerging from our investigations utilizes the four-state conformational landscape where mutations that stabilize closed states increase the rigidity of protease. In contrast, those mutations that lead to multidrug resistance modulate the conformational landscape to stabilize the open-like states, destabilize the closed state, and increase the overall protein backbone dynamics [12,19]. The fractional occupancy, *f*_i_, of each state can be reflective of the relative thermodynamic stability Gibbs free energy, ∆*G*, where the more populated the state, the more stable it is given by ∆*G* = −*RT* ln*f*_i_.

The investigations into these DRV-resistant constructs uphold our earlier findings and lend further support to the conformational selection hypothesis. Interestingly, our earlier studies on the accumulation of mutations in response to NFV resulted in an enzyme with catalytic activity comparable to WT but resistant to >3 inhibitors. For DRV resistance, we note that these accumulated mutations do not result in an enzyme with activity comparable to WT. Perhaps this arises because the sequences we investigated are not clinical isolates but rather generated from commonly seen DRV primary and secondary mutations. An additional explanation may be that because DRV was designed to closely mimic the substrate envelop [32] such that evolving resistance would be difficult, it is reasonable that mutations that destabilize DRV binding may also compromise substrate binding, which is a result that we see in our DEER data and is reflected in published kinetic studies of others [31].

## Figures and Tables

**Table 1:** DEER distance profiles for HIV-1 DRV-resistant proteases and PI-naïve subtype B.

	Unbound		CaP2		DRV	
HIV-1 Constructs	Most Probable Distance (Å) (Error ± 0.2)	Average Distance (Å) (Error ± 0.2)	Most Probable Distance (Å) (Error ± 0.2)	Average Distance (Å) (Error ± 0.2)	Most Probable Distance (Å) (Error ± 0.2)	Average Distance (Å) (Error ± 0.2)
DRV1	40.7	34.6	36.8	35.9	38.7	34.9
DRV2	39.1	36.9	39.1	36.0	40.5	37.1
DRV3	32.2	31.6	32.6	33.3	32.6	32.7
DRV4	30.6	34.2	32.3	32.5	37.4	33.0
DRV5	36.9	33.8	37.3	35.2	34.5	33.5
DRV6	34.4	32.4	34.0	32.3	34.5	33.0
PI-naïve B ^1^	36.2	36.2	33.1	33.9	33.2	33.6

^1^ Data taken from [20].

**Table 2:** Summary of the fractional occupancy of the four nominal states from DEER population analysis.

Constructs	States	Relative Populations (±5%)			
		Curled/Tucked	Closed	Semi-Open	Wide-Open
**DRV1**	unbound	31	21	13	35
	CaP2	18	20	41	21
	DRV	26	20	35	19
DRV2	unbound	15	21	37	27
	CaP2	9	30	37	24
	DRV	9	19	38	34
DRV3	unbound	27	61	5	7
	CaP2	7	77	10	6
	DRV	0	100	0	0
DRV4	unbound	37	26	22	15
	CaP2	41	31	17	11
	DRV	36	30	20	14
DRV5	unbound	19	30	40	11
	CaP2	13	38	35	14
	DRV	20	41	25	14
DRV6	unbound	19	44	37	0
	CaP2	18	61	21	0
	DRV	17	52	31	0
B ^1^	Unbound	0	3	90	7
	CaP2	0	80	16	4
	DRV	0	87	13	0

^1^ Data taken from [20]

## Supplementary Materials

The following are available online at https://www.mdpi.com/1999-4915/12/11/1275/s1, Table S1: Summary of expected and observed mass for MTSL labeled HIV-1 PR constructs determined from mass spectrometry. Figure S1: Amino acid sequences of constructs utilized. Figure S2:100G CW X-band EPR spectra for DRV3 HIV-1 PR (A) as a function of solution pH in 20 mM D_3_-NaOAc/D_2_O with 30% *v/v* D_8_-glycerol compared to spectrum obtained for WT (Bsi) and (B) at pH 5.0 with DRV addition. Figure S3: Stack plot of 100G CW X-band EPR spectra for unbound HIV-1 PR DRV1 showing how pH alters spectra, which is inferred as sample homogeneity. Figure S4: DLS results as a function of pH for DRV3 and DRV1 with and without DRV. Figure S5: DEER data for apo HIV-1 PR DRV1 pH 2.8. Figure S6: DEER data for CaP2-bound HIV-1 PR DRV1 pH 5.0. Figure S7: DEER data for DRV-bound HIV-1 PR DRV1 pH 5.0. Figure S8: DEER data for apo HIV-1 PR DRV2, pH 5.0. Figure S9: DEER data for CaP2-bound HIV-1 PR DRV2, pH 5.0. Figure S10: DEER data for DRV-bound HIV-1 PR DRV2, pH 5.0. Figure S11: DEER data for apo HIV-1 PR DRV3, pH 5.0. Figure S12: DEER data for apo HIV-1 PR DRV3, pH 3.0. Figure S13: DEER data for CaP2-bound HIV-1 PR DRV3, pH 5.0. Figure S14: DEER data for DRV-bound HIV-1 PR DRV3, pH 5.0. Figure S15: DEER data for apo HIV-1 PR DRV4, pH 5.0. Figure S16: DEER data for CaP2-bound HIV-1 PR DRV4, pH 5.0. Figure S17: DEER data for DRV-bound HIV-1 PR DRV4, pH 5.0. Figure S18: DEER data for apo HIV-1 PR DRV5, pH 5.0. Figure S19: DEER data for CaP2-bound HIV-1 PR DRV5, pH 5.0. Figure S20: DEER data for DRV-bound HIV-1 PR DRV5, pH 5.0. Figure S21: DEER data for apo HIV-1 PR DRV6, pH 5.0. Figure S22: DEER data for CaP2-bound HIV-1 PR DRV6, pH 5.0. Figure S23: DEER data for DRV-bound HIV-1 PR DRV6, pH 5.0. Table S2: DEER population analysis via Gaussian reconstruction. Table S3: Relative Populations of Conformational States Determined from DEER Analysis. Table S4: Z-test for Evaluating the Difference in the Semi-open Population Relative to Subtype B. Table S5: Z-test for Evaluating the Difference in the Open-Like = Wide-Open+ Curled Populations Relative to Subtype B. Table S6: Z-test for Evaluating the Difference in the Closed Populations Relative to Subtype B Figure S24: Analysis of significance of population differences for conformational sampling of DRV constructs relative to Subtype B.
