# Supplementary material for: Darunavir-Resistant HIV-1 Protease Constructs Uphold a Conformational Selection Hypothesis for Drug Resistance
Source: Viruses. 2020 Nov 8;12(11):1275. doi: 10.3390/v12111275 (PMC7695139; doi:10.3390/v12111275)
Supplement: Supplementary file 1 [file viruses-12-01275-s001.pdf]

Darunavir Resistant HIV-1 Protease Constructs Uphold a Conformational Selection Hypothesis for Drug Resistance

Zhanglong Liu [1,2], Trang T. Tran [1], Linh Pham [1,3], Lingna Hu [1], Kyle Bentz [1,4], Daniel A. Savin [1] and Gail E. Fanucci [1,*]

[1] Department of Chemistry, University of Florida, Gainesville, FL 32611, USA; zhanglong.liu@gmail.com (Z.L.); trangtran@ufl.edu (T.T.T.); linhpham@tamuct.edu (L.P.); lnhu39@chem.ufl.edu (L.H.); kcbentz@chem.ufl.edu (K.B.); savin@chem.ufl.edu (D.A.S.)
[2] LinkedIn, Mountain View, CA, 94043, USA
[3] Department of Science and Mathematics, Texas A&M University—Central Texas, Killeen, TX 76549, USA
[4] Department of Chemistry and Biochemistry, University of California, San Diego, La Jolla, CA 92093, USA
* Correspondence: fanucci@chem.ufl.edu; Tel.: +352-392-2345; Fax: +352-392-0872

Supplementary Information contains the following information

1. Mass spectrometry analysis ....Page 2
2. HIV-1 PR amino acid sequence tables... Page 3
3. CW EPR investigations for DRV1 and DRV3..Page 4
4. Dynamic Light Scattering Analysis of DRV1 and DRV3...................................... Pages 5 -6
5. Details of DEER data analysis via TKR and DEERConstruct................................Pages 7-24
6. Details of Population Analysis Significance Z-Test...Pages 25-29

1. Mass Spectrometry Analysis

Mass spectrometry is performed on all spin-labeled samples after final purification steps to ensure near complete labeling and appropriate protein mass. Data was collected on an Agilent 6220 ESI TOF (Santa Clara, CA) mass spectrometer equipped with an electrospray source operated in positive ion mode. Agilent ESI Low Concentration Tuning Mix was used for mass calibration for a calibration range of m/z 100 - 2000. Samples were prepared in a solution containing acidified acetonitrile (0.5% formic acid) and 1 μL was injected into the electrospray source at a rate of 100 ml min^{-1}. Optimal conditions were capillary voltage 4000 V, source temperature 350°C and a cone voltage of 60 V. The TOF analyzer was scanned over an appropriate m/z range with a 1 s integration time. Data was acquired in continuum mode until acceptable averaged data was obtained. ESI results were collected for all samples and complete spin labeling of proteins was confirmed with correctly anticipated masses before proceeding to DEER data collection.

Table 1. Summary of expected and observed mass for MTSL labeled HIV-1 PR constructs determined from mass spectrometry.

HIV-1 PR	Theoretical MW of PR (Da)	Theoretical MW of MTSL-bound PR (Da)	Observed MW of MTSL-bound PR (Da)
DRV1	10755.66	10939.94	10940.10
DRV2	10695.53	10878.81	10880.3
DRV3	10747.69	10931.97	10932.57
DRV4	10745.50	10929.78	10930.10
DRV5	10824.59	11008.87	11009.10
DRV6	10605.35	10789.63	10789.80

2. HIV-1 PR amino acid sequence summary

HIV-1 PR sequence summary. The sequence of PI-naïve subtype B is given as the reference. This sequence is based off the LAI sequence with the following substitutions shown in bold: C67A, C95A, Q7K, L33I, L63I, D25N and K55C. DRV1-6 sequences are shown with drug pressure selected substitutions shown in bold and underlined.

	10 20 30 40 50
**Subtype B**	PQITLWKRPL VTIKIGGQLK EALLNTGADD TVIEEMSLPG RWKPKMIGGI
**DRV1**	PQITLWQRPL VVVKVGGQLM EALLNTGADD TIFEEMNLPG RWTPKMIGGI
**DRV2**	PQITLWQRPL VTVKIGGQLR EALLNTGADD TIFEDISLPG KWTPKMVGGI
**DRV3**	PQITLWQRPF VTVKIGGQLK EALLNTGADN TVLEEMSLPG RWKPIMIGGL
**DRV4**	PQITLWQRPI VTVRIGGQLK EALLNTGADD TIFEEMSLPG RWTPKIVGGI
**DRV5**	PQITLWQRPI VTVKIEGQLK EALLNTGADD TVFEELTLSG RWKPRLIGGI
**DRV6**	PQITLWQRPI VTVKVGGQLR EALLNTGADD TVFNDISLPG RWTPKMVGGL
	**60 70 80 90**
**Subtype B**	GGFICVRQYD QIIIEIAGHK AIGTVLVGPT PVNIIGRNLL TQIGATLNF
**DRV1**	GGFLCVRQYD QVPIEIAGHK VVSTVLIGPT PLNVIGRNVM TQIGATLNF
**DRV2**	GGFMCVRQYD QVVIEIAGHK VTSPVLVGPT PLNIIGRNVL TQLGATLNF
**DRV3**	GGFICVRQYD QIPIEIAGHK IIGTVLIGPT PVNIIGRDLL TQIGATLNF
**DRV4**	GGFLCVRQYD QVPIEIAGHK TTTTVLIGST PVNVIGRNLM TQIGATLNF
**DRV5**	GGFVCVRQYD QVPIEIAGHK VIDTVLVGPT PTNVIGRNVM TQLGATLNF
**DRV6**	GGLICVREYD QVPIEFAGHK VIGTVLIGPT PANVIGRNVL TQIGATLNF

Figure 1. Sequence alignment of constructs studied here.

3. CW EPR spectra

CW EPR spectra are recorded for each sample prior to and after DEER analysis to ensure sample quality. We noted for DRV1 and DRV3 that at pH = 5.0 the CW lineshapes differed dramatically than other samples, in particular PI-naive subtype B, and appeared similar to spectra observed previously when exploring the impact of salt concentration on WT (Bs).[1] We attribute this broadened spectrum to some form of solution aggregate that is soluble (see DLS data below). Note, solution was not cloudy upon inspection, so no precipitate was forming, however at higher pH values, broadened spectra were obtained. This effect is "reversable" because in SI-2 (B) spectra show that upon addition of DRV (at pH 5.0) the spectrum of DRV3+DRV resembles that expected for well-behaved dimer in solution (spectrum of Bsi pH 5.0). Also note, our lab has performed a series of solution NMR experiments upon various HIV-1 PR constructs [8-10] so we know how to prepare a homogeneous well behaved sample and know that the spectra shown in (A) are representative of some solution aggregate.

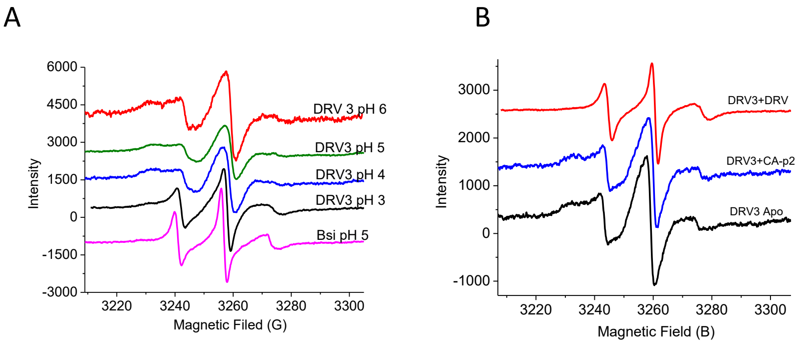

Figure 2. 100G CW X-band EPR spectra for DRV3 HIV-1 PR (A) as a function of solution pH in20 mM D_3-NaOAc/D_2O, with 30% v/v D_8-glycerol compared to spectrum obtained for WT (Bsi) and (B) at pH 5.0 with DRV addition . Spectra are vertically offset for clarity.

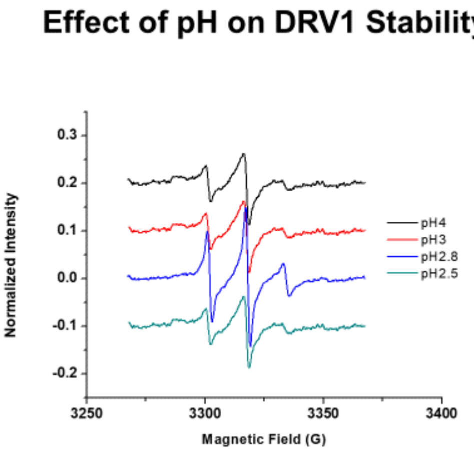

Figure 3. Stack plot of 100G CW X-band EPR spectra for unbound HIV-1 PR DRV1 showing how pH alters spectra which is inferred as sample homogeneity. Best spectrum was obtained at pH 2.8, so DEER data for this construct was performed at this pH. [1.]

4. Dynamic Light Scattering

Dynamic light scattering (DLS) measurements were performed on an ALV/CGS-3 four-angle, compact goniometer system (Langen, Germany), equipped with a 22 mW HeNe linear polarized laser operating at a wavelength of λ = 632.8 nm. Fluctuations in the scattering intensity were measured via a ALV/LSE-5004 multiple tau digital correlator and analyzed via the intensity autocorrelation function.

$$g^2(\tau) = \frac{\langle I(t+\tau)I(t)\rangle}{\langle I\rangle^2}$$

where $\langle I\rangle$ is the average scattering intensity and $I(t)$ is the scattering intensity at time t, and τ is the delay time. The correlation functions at 90° were deconvoluted using a regularized inverse Laplace transform (CONTIN analysis), which yields a distribution of decay rates, Γ_i, by

$$g^1(q,t) = \int_0^{\infty} G(\Gamma_i) exp(-\Gamma_i t) d\Gamma$$

where $g^1(q,t)$ is the normalized electric field autocorrelation function. The mutual diffusion coefficient, D_m, for a particular species in the distribution is determined by $D_{m,i} = \Gamma_i/q^2$, from which the hydrodynamic radii, R_h, of the corresponding particles can be determined using the Stokes-Einstein relationship,

$$D_m \approx D_o = \frac{k_B T}{6\pi\eta_s R_h}$$

where k_B is the Boltzmann constant, T is the absolute temperature, and η_s is the solvent viscosity. Light scattering measurements were performed at 25 °C. Samples were diluted to 0.1 mg/mL in water, passed through 0.45 μm poly(vinylidene fluoride) syringe filters into precleaned borosilicate tubes for analysis.

DLS data reveal that larger aggregates are forming in solution for DRV1 and DRV3 at pH 5.0; where previous DEER, CW EPR and NMR investigations have been performed for various HIV-1PR constructs. DLS results at lowered pH (4 and 3 for DRV3, and 2.8 for DRV1) give DLS results similar to those obtained for Subtype B. DLS also shows that at pH 5.0; the addition of inhibitor (DRV) to DRV3 shifts the size distribution to a profile seen for subtype B with DRV. This results is consistent with CW EPR results showing the narrowed line shape observed upon addition of inhibitor. Although at pH 5.0 the addition of DRV to DRV1 shifts the profile to smaller sizes (Fig SI-4D), the size distribution is still larger than that observed with subtype B. Upon dropping pH to 2.8 the size distribution of unbound DRV1 now matches that of subtypeB, the distribution profile upon addition of DRV is altered from what has been observed previously. All DEER data for addition of DRV and CaP2 were performed at pH 5.0 to help aid in protein stability.

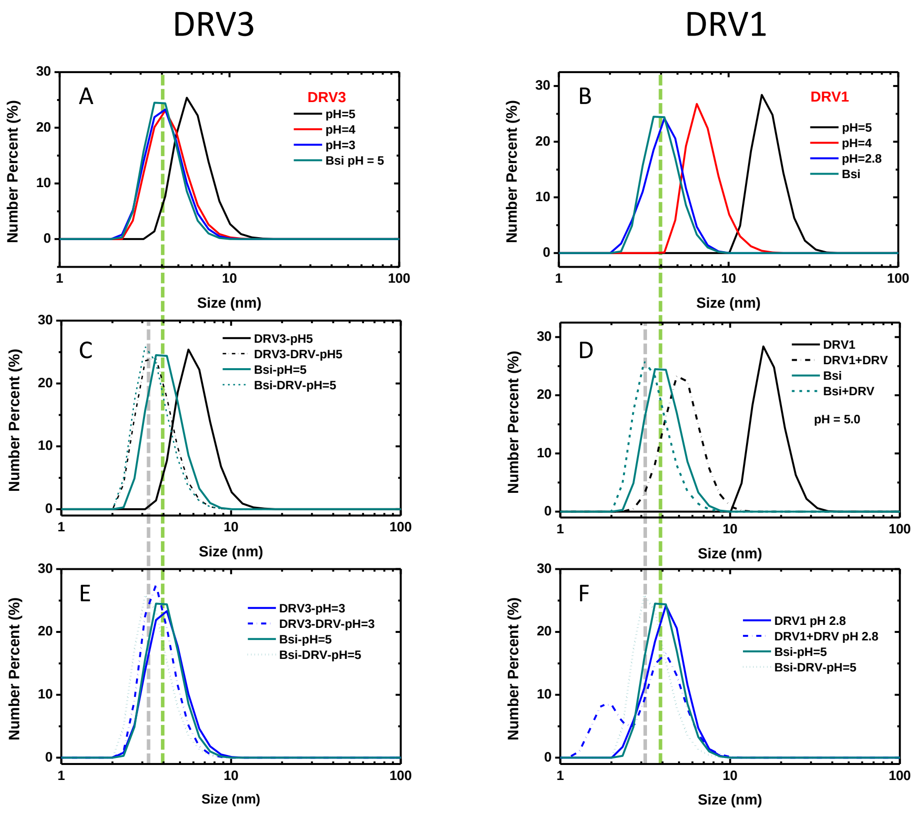

Figure S4. DLS results as a function of pH for (A) DRV3 and (B)DRV1, results for individual DRV constructs with and without DRV at pH 5.0 for (C) DRV3 and (D) DRV1, and (E) at pH 3.0 for DRV3 and (F) pH 2.8 for DRV1. A-F also show results for PI niave-subtype B (labeled Bsi) for comparison. Dashed lines are guides for the eyes.

5. DEER Data Analyses and Summaries

The summary of relative percentages of subtype B has been published previously.[2, 3] All DEER data was processed to generate a background-corrected dipolar modulation curve and a distance profile using DeerAnalysis2019.[4] The validity of each population contributing less than 20% to the total population was tested by suppressing the population of interest, generating a theoretical echo curve, and comparing the generated theoretical echo curve to the background-corrected echo curve using DEERconstruct program.[5-8] Results are shown in Supporting Information Figures SI-5 to SI-23 for each construct investigated here in unbound form, upon addition of DRV and CaP2. For DRV1 and DRV3 effects of sample pH are also shown.

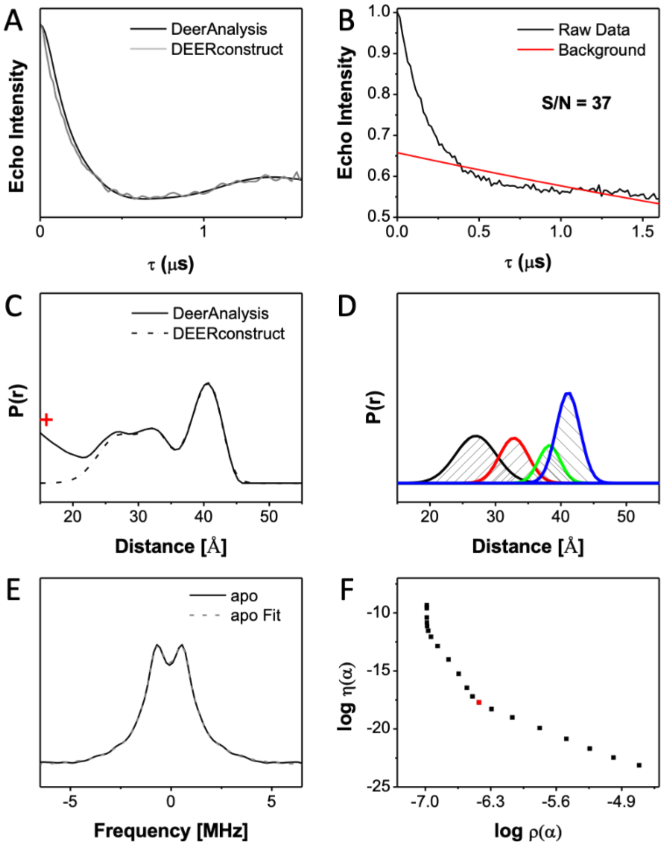

Figure S5. DEER data for apo HIV-1 PR DRV1 pH 2.8, **A**) Background corrected dipolar evolution curve after the long pass filter in DeerAnalysis (black) and the simulated curve from DEERconstruct (gray); **B**) Raw dipolar evolution curve and background, the signal to noise ratio (S/N) is shown inset, where the signal is the DEER modulation depth and the noise is 2 times of the standard deviation of the noise curve; **C**) The corresponding distance profile generated via TKR analysis by DeerAnalysis (black) and the theoretical curve generated from the Gaussian reconstruction by DEERconstruct (gray), "+" indicates that the peak is presumed to be an artifact of processing as it is near the lower limit of the generally accepted range that is measurable using DEER; **D**) The individual Gaussian functions used in the reconstruction; **E**) Frequency domain spectrum; **F**) L-curve derived from TKR fit to obtain the optimal regulation parameter, the optimal regulation parameter is plot in red.

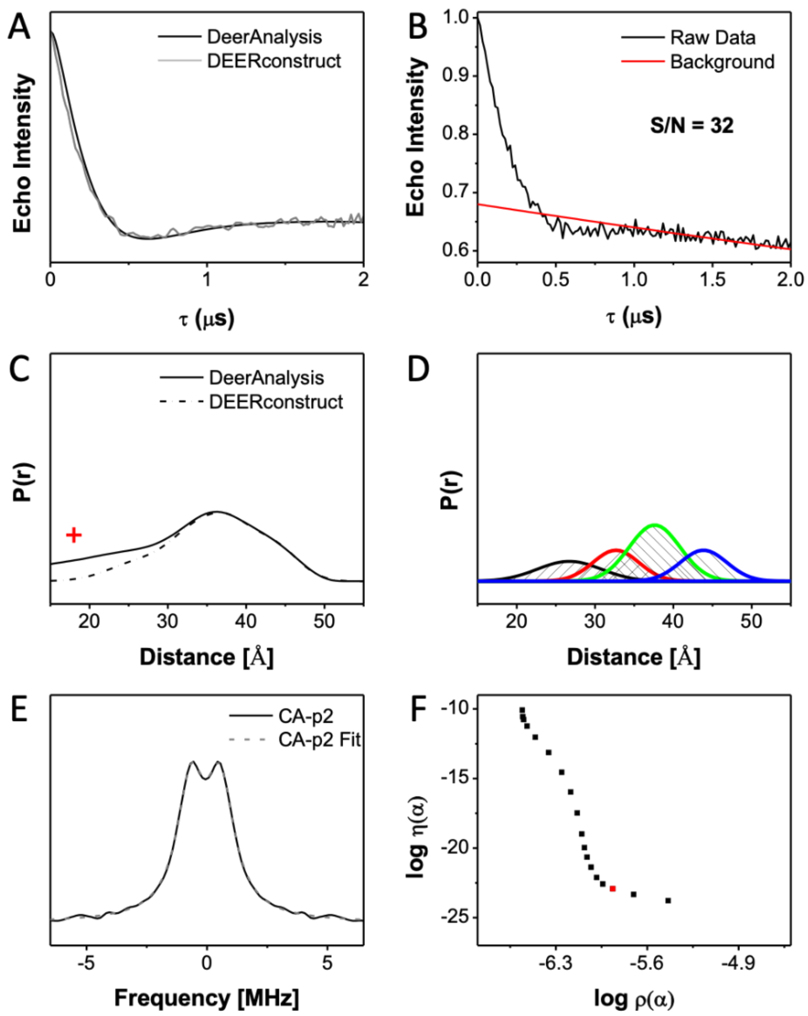

Figure S6. DEER data for CaP2-bound HIV-1 PR DRV1 pH 5.0, **A**) Background corrected dipolar evolution curve after the long pass filter in DeerAnalysis (black) and the simulated curve from DEERconstruct (gray); **B**) Raw dipolar evolution curve and background, the signal to noise ratio (S/N) is shown inset, where the signal is the DEER modulation depth and the noise is 2 times of the standard deviation of the noise curve; **C**) The corresponding distance profile generated via TKR analysis by DeerAnalysis (black) and the theoretical curve generated from the Gaussian reconstruction by DEERconstruct (gray), "+" indicates that the peak is presumed to be an artifact of processing as it is near the lower limit of the generally accepted range that is measurable using DEER; **D**) The individual Gaussian functions used in the reconstruction; **E**) Frequency domain spectrum; **F**) L-curve derived from TKR fit to obtain the optimal regulation parameter, the optimal regulation parameter is plot in red.

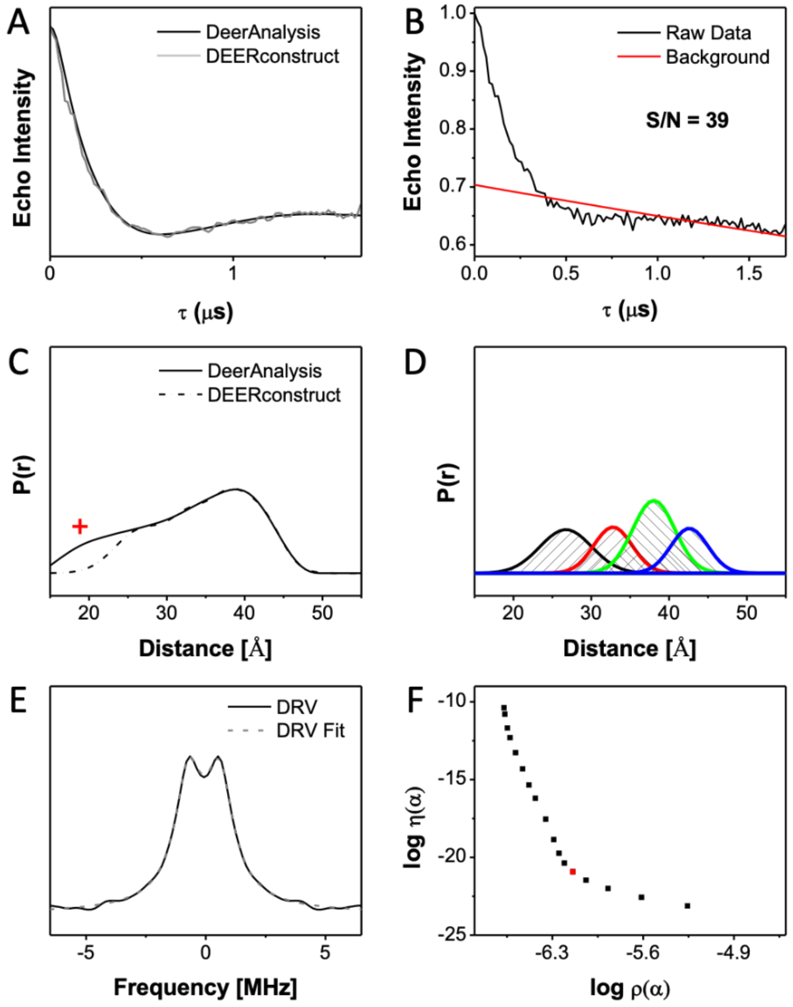

Figure S7. DEER data for DRV-bound HIV-1 PR DRV1 pH 5.0, **A**) Background corrected dipolar evolution curve after the long pass filter in DeerAnalysis (black) and the simulated curve from DEERconstruct (gray); **B**) Raw dipolar evolution curve and background, the signal to noise ratio (S/N) is shown inset, where the signal is the DEER modulation depth and the noise is 2 times of the standard deviation of the noise curve; **C**) The corresponding distance profile generated via TKR analysis by DeerAnalysis (black) and the theoretical curve generated from the Gaussian reconstruction by DEERconstruct (gray), "+" indicates that the peak is presumed to be an artifact of processing as it is near the lower limit of the generally accepted range that is measurable using DEER; **D**) The individual Gaussian functions used in the reconstruction; **E**) Frequency domain spectrum; **F)** L-curve derived from TKR fit to obtain the optimal regulation parameter, the optimal regulation parameter is plot in red.

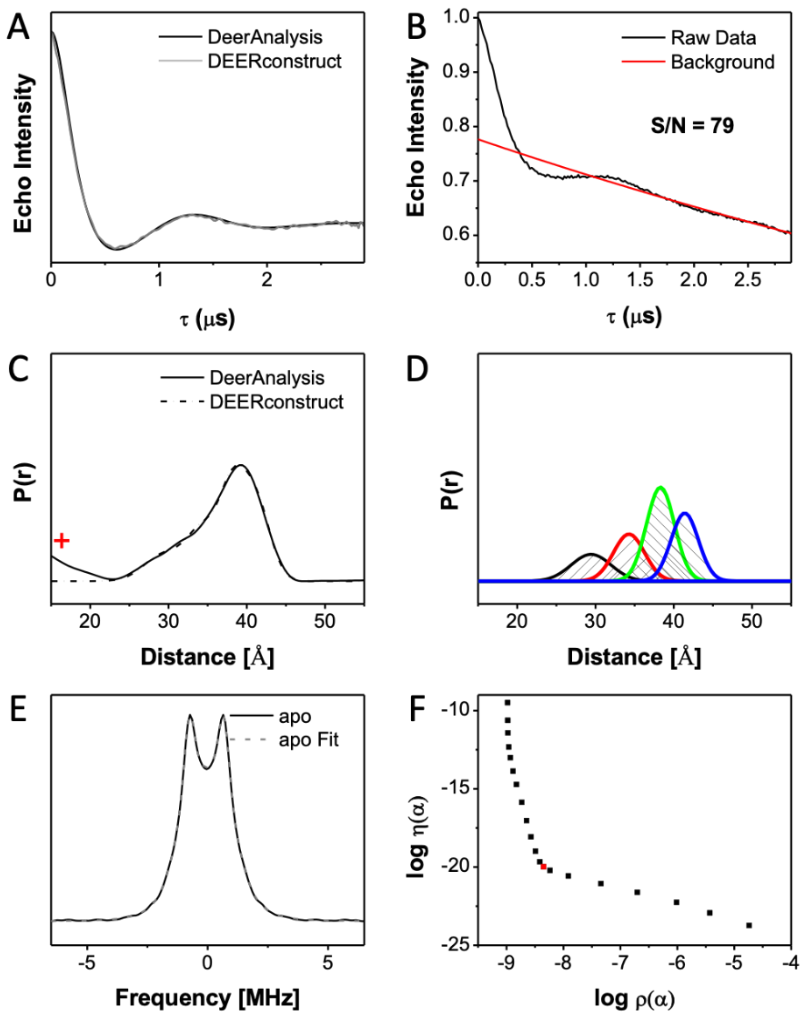

Figure S8. DEER data for apo HIV-1 PR DRV2, pH 5.0 **A**) Background corrected dipolar evolution curve after the long pass filter in DeerAnalysis (black) and the simulated curve from DEERconstruct (gray); **B**) Raw dipolar evolution curve and background, the signal to noise ratio (S/N) is shown inset, where the signal is the DEER modulation depth and the noise is 2 times of the standard deviation of the noise curve; **C**) The corresponding distance profile generated via TKR analysis by DeerAnalysis (black) and the theoretical curve generated from the Gaussian reconstruction by DEERconstruct (gray), "+" indicates that the peak is presumed to be an artifact of processing as it is near the lower limit of the generally accepted range that is measurable using DEER; **D**) The individual Gaussian functions used in the reconstruction; **E**) Frequency domain spectrum; **F**) L-curve derived from TKR fit to obtain the optimal regulation parameter, the optimal regulation parameter is plot in red.

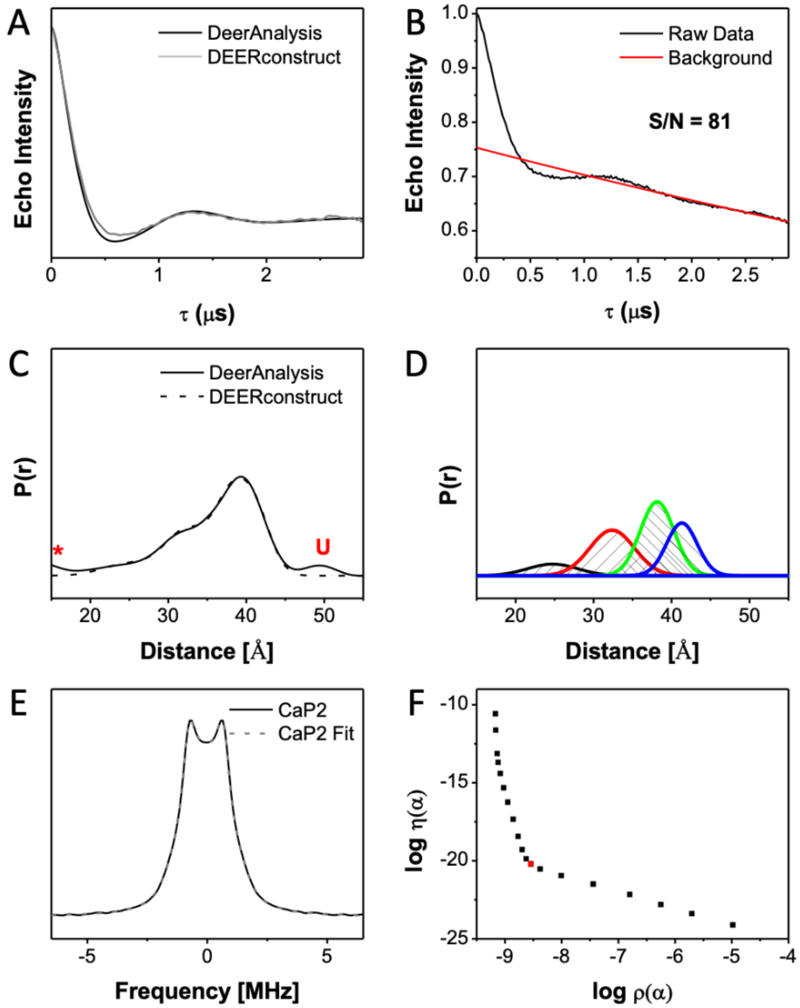

Figure S9. DEER data for CaP2-bound HIV-1 PR DRV2, pH 5.0 **A**) Background corrected dipolar evolution curve after the long pass filter in DeerAnalysis (black) and the simulated curve from DEERconstruct (gray); **B**) Raw dipolar evolution curve and background, the signal to noise ratio (S/N) is shown inset, where the signal is the DEER modulation depth and the noise is 2 times of the standard deviation of the noise curve; **C**) The corresponding distance profile generated via TKR analysis by DeerAnalysis (black) and the theoretical curve generated from the Gaussian reconstruction by DEERconstruct (gray), asterisks indicate that peaks are within the suppression range, "U" indicates the unsigned peak, which is far longer distance than the 41~42 angstrom wide-open states; **D**) The individual Gaussian functions used in the reconstruction; **E**) Frequency domain spectrum; **F**) L-curve derived from TKR fit to obtain the optimal regulation parameter, the optimal regulation parameter is plot in red.

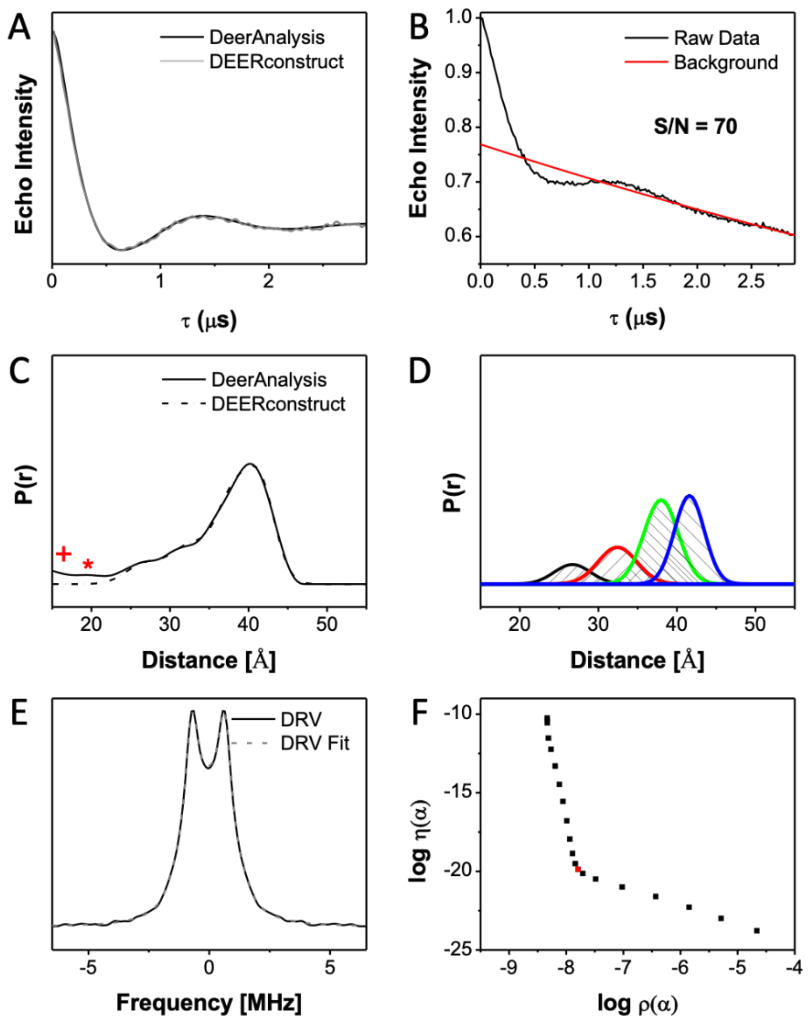

Figure S10. DEER data for DRV-bound HIV-1 PR DRV2, pH 5.0 **A**) Background corrected dipolar evolution curve after the long pass filter in DeerAnalysis (black) and the simulated curve from DEERconstruct (gray); **B**) Raw dipolar evolution curve and background, the signal to noise ratio (S/N) is shown inset, where the signal is the DEER modulation depth and the noise is 2 times of the standard deviation of the noise curve; **C**) The corresponding distance profile generated via TKR analysis by DeerAnalysis (black) and the theoretical curve generated from the Gaussian reconstruction by DEERconstruct (gray), asterisks indicate that peaks are within the suppression range, "+" indicates that the peak is presumed to be an artifact of processing as it is near the lower limit of the generally accepted range that is measurable using DEER; **D**) The individual Gaussian functions used in the reconstruction; **E**) Frequency domain spectrum; **F**) L-curve derived from TKR fit to obtain the optimal regulation parameter, the optimal regulation parameter is plot in red.

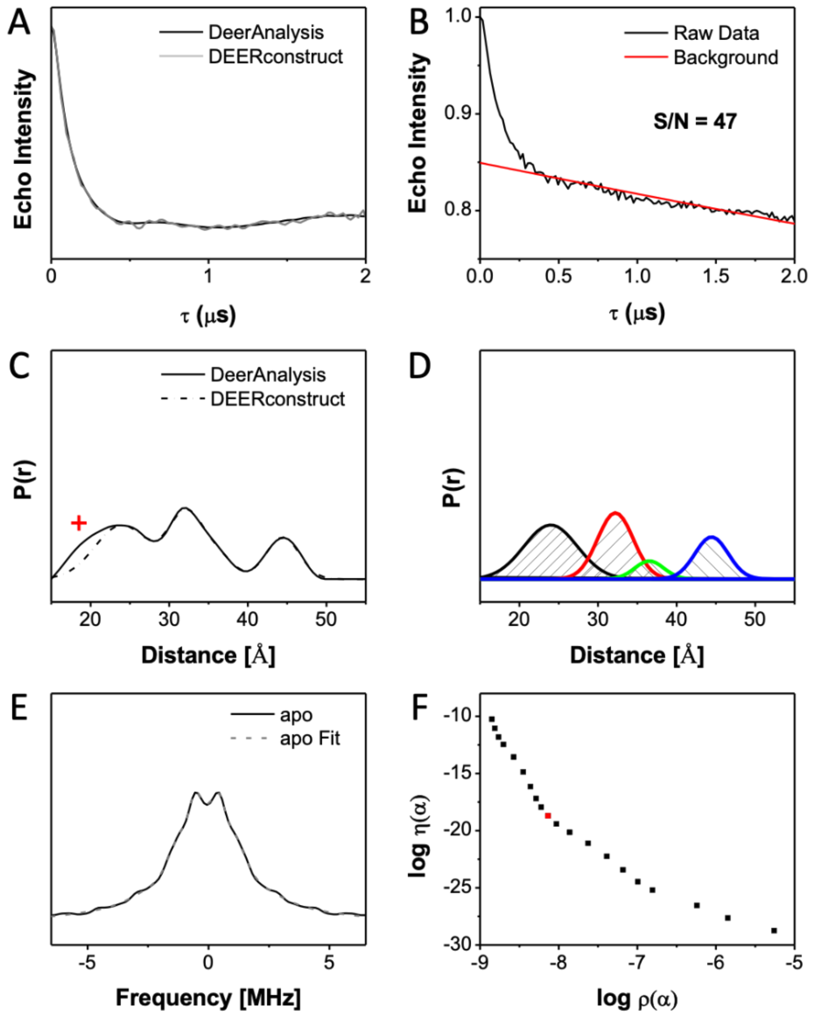

Figure S11. DEER data for apo HIV-1 PR DRV3 at pH 5.0, **A**) Background corrected dipolar evolution curve after the long pass filter in DeerAnalysis (black) and the simulated curve from DEERconstruct (gray); **B**) Raw dipolar evolution curve and background, the signal to noise ratio (S/N) is shown inset, where the signal is the DEER modulation depth and the noise is 2 times of the standard deviation of the noise curve; **C**) The corresponding distance profile generated via TKR analysis by DeerAnalysis (black) and the theoretical curve generated from the Gaussian reconstruction by DEERconstruct (gray), "+" indicates that the peak is presumed to be an artifact of processing as it is near the lower limit of the generally accepted range that is measurable using DEER; **D**) The individual Gaussian functions used in the reconstruction; **E**) Frequency domain spectrum; **F**) L-curve derived from TKR fit to obtain the optimal regulation parameter, the optimal regulation parameter is plot in red.

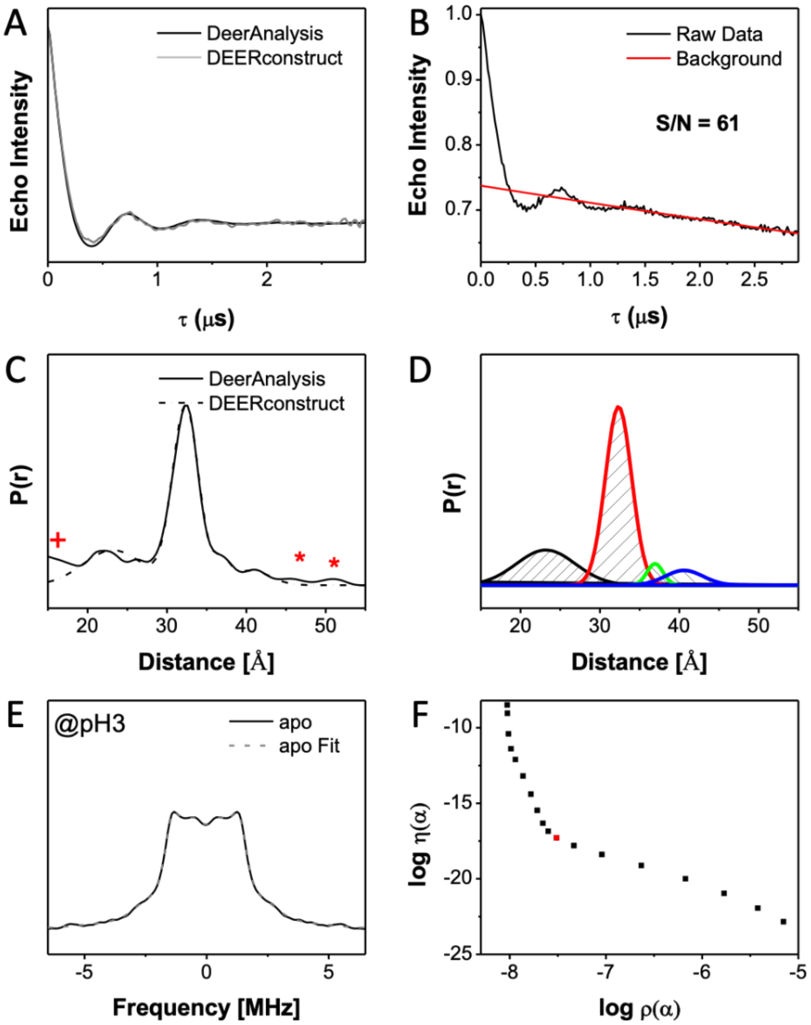

Figure S12. DEER data for apo HIV-1 PR DRV3 at pH 3, **A**) Background corrected dipolar evolution curve after the long pass filter in DeerAnalysis (black) and the simulated curve from DEERconstruct (gray); **B**) Raw dipolar evolution curve and background, the signal to noise ratio (S/N) is shown inset, where the signal is the DEER modulation depth and the noise is 2 times of the standard deviation of the noise curve; **C**) The corresponding distance profile generated via TKR analysis by DeerAnalysis (black) and the theoretical curve generated from the Gaussian reconstruction by DEERconstruct (gray), asterisks indicate that peaks are within the suppression range, "+" indicates that the peak is presumed to be an artifact of processing as it is near the lower limit of the generally accepted range that is measurable using DEER; **D**) The individual Gaussian functions used in the reconstruction; **E**) Frequency domain spectrum; **F**) L-curve derived from TKR fit to obtain the optimal regulation parameter, the optimal regulation parameter is plot in red.

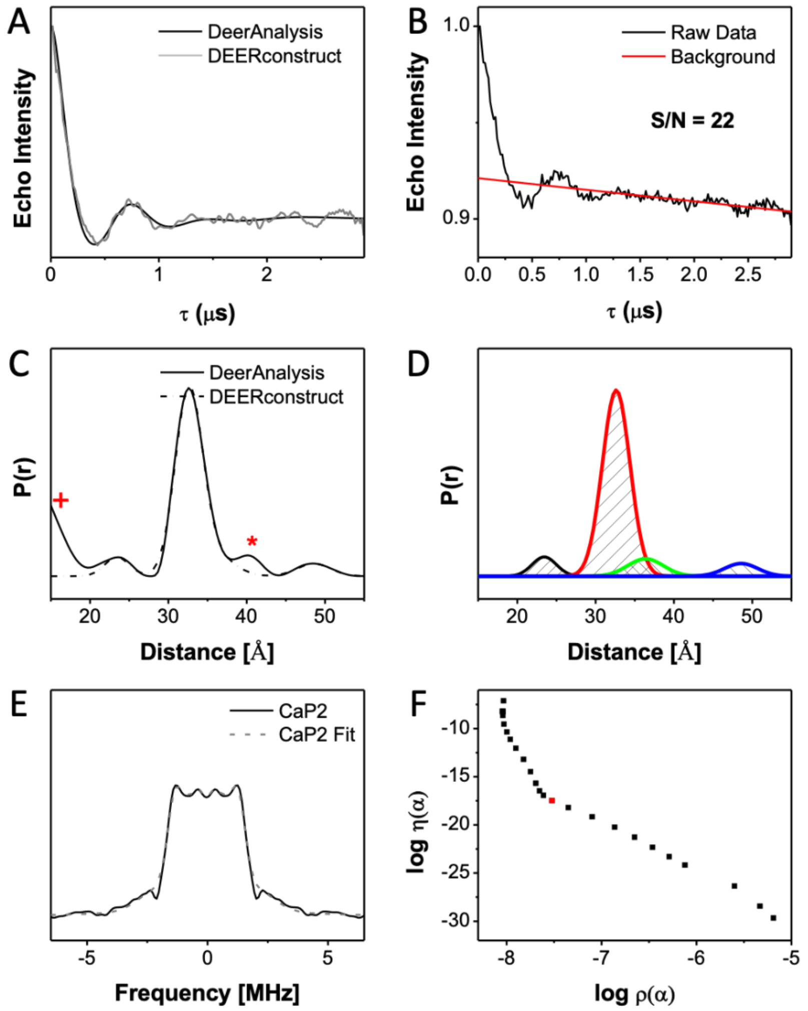

Figure S13. DEER data for CaP2-bound HIV-1 PR DRV3, pH 5.0 **A**) Background corrected dipolar evolution curve after the long pass filter in DeerAnalysis (black) and the simulated curve from DEERconstruct (gray); **B**) Raw dipolar evolution curve and background, the signal to noise ratio (S/N) is shown inset, where the signal is the DEER modulation depth and the noise is 2 times of the standard deviation of the noise curve; **C**) The corresponding distance profile generated via TKR analysis by DeerAnalysis (black) and the theoretical curve generated from the Gaussian reconstruction by DEERconstruct (gray), asterisks indicate that peaks are within the suppression range, "+" indicates that the peak is presumed to be an artifact of processing as it is near the lower limit of the generally accepted range that is measurable using DEER; **D**) The individual Gaussian functions used in the reconstruction; **E**) Frequency domain spectrum; **F**) L-curve derived from TKR fit to obtain the optimal regulation parameter, the optimal regulation parameter is plot in red.

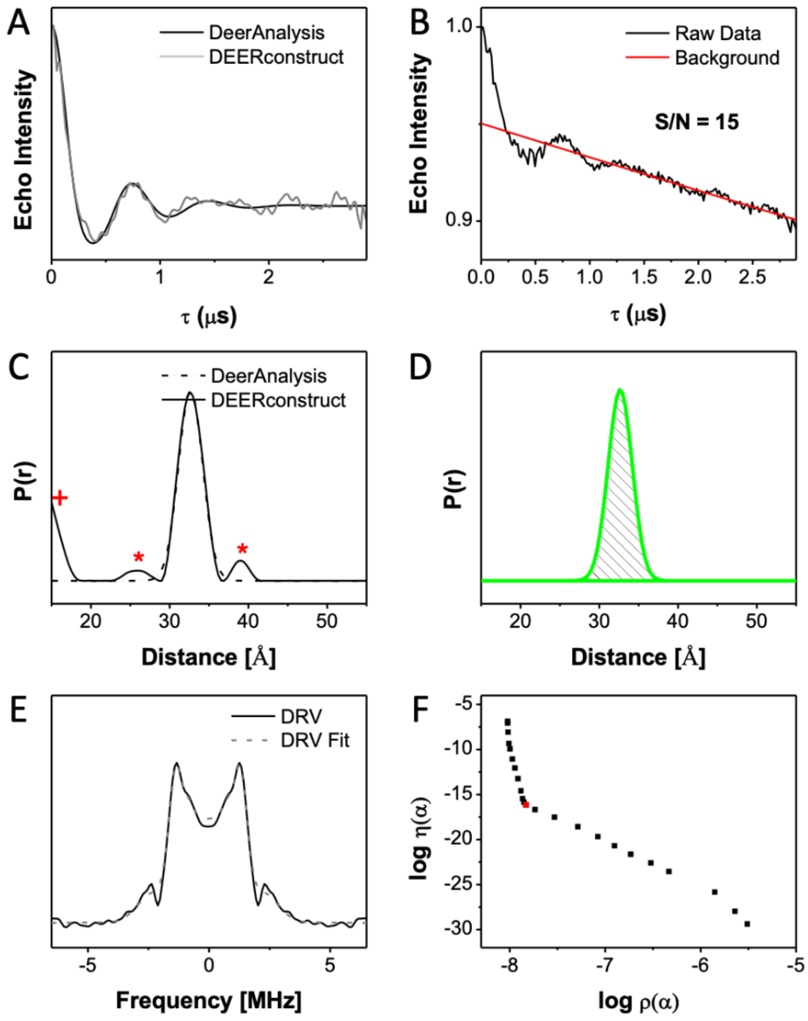

Figure S14. DEER data for DRV-bound HIV-1 PR DRV3, pH 5.0 **A**) Background corrected dipolar evolution curve after the long pass filter in DeerAnalysis (black) and the simulated curve from DEERconstruct (gray); **B**) Raw dipolar evolution curve and background, the signal to noise ratio (S/N) is shown inset, where the signal is the DEER modulation depth and the noise is 2 times of the standard deviation of the noise curve; **C**) The corresponding distance profile generated via TKR analysis by DeerAnalysis (black) and the theoretical curve generated from the Gaussian reconstruction by DEERconstruct (gray), asterisks indicate that peaks are within the suppression range, "+" indicates that the peak is presumed to be an artifact of processing as it is near the lower limit of the generally accepted range that is measurable using DEER; **D**) The individual Gaussian functions used in the reconstruction; **E**) Frequency domain spectrum; **F**) L-curve derived from TKR fit to obtain the optimal regulation parameter, the optimal regulation parameter is plot in red.

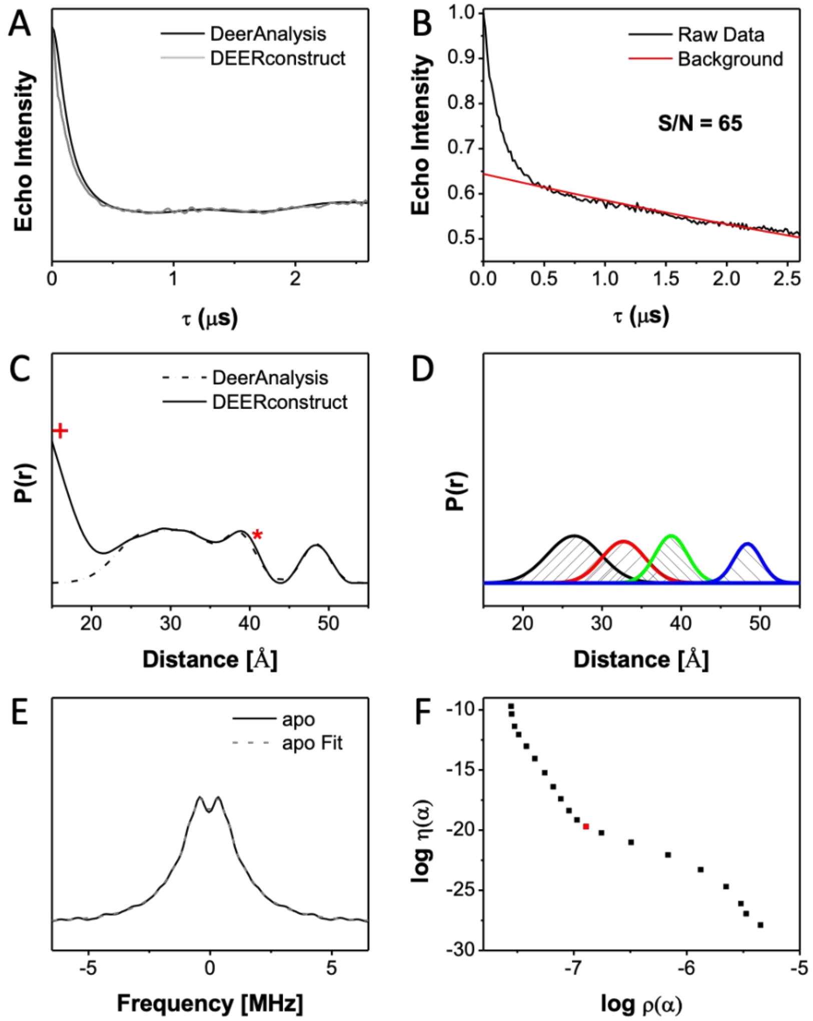

Figure S15. DEER data for apo HIV-1 PR DRV4, pH 5.0 **A**) Background corrected dipolar evolution curve after the long pass filter in DeerAnalysis (black) and the simulated curve from DEERconstruct (gray); **B**) Raw dipolar evolution curve and background, the signal to noise ratio (S/N) is shown inset, where the signal is the DEER modulation depth and the noise is 2 times of the standard deviation of the noise curve; **C**) The corresponding distance profile generated via TKR analysis by DeerAnalysis (black) and the theoretical curve generated from the Gaussian reconstruction by DEERconstruct (gray), asterisks indicate that peaks are within the suppression range, "+" indicates that the peak is presumed to be an artifact of processing as it is near the lower limit of the generally accepted range that is measurable using DEER; **D**) The individual Gaussian functions used in the reconstruction; **E**) Frequency domain spectrum; **F**) L-curve derived from TKR fit to obtain the optimal regulation parameter, the optimal regulation parameter is plot in red.

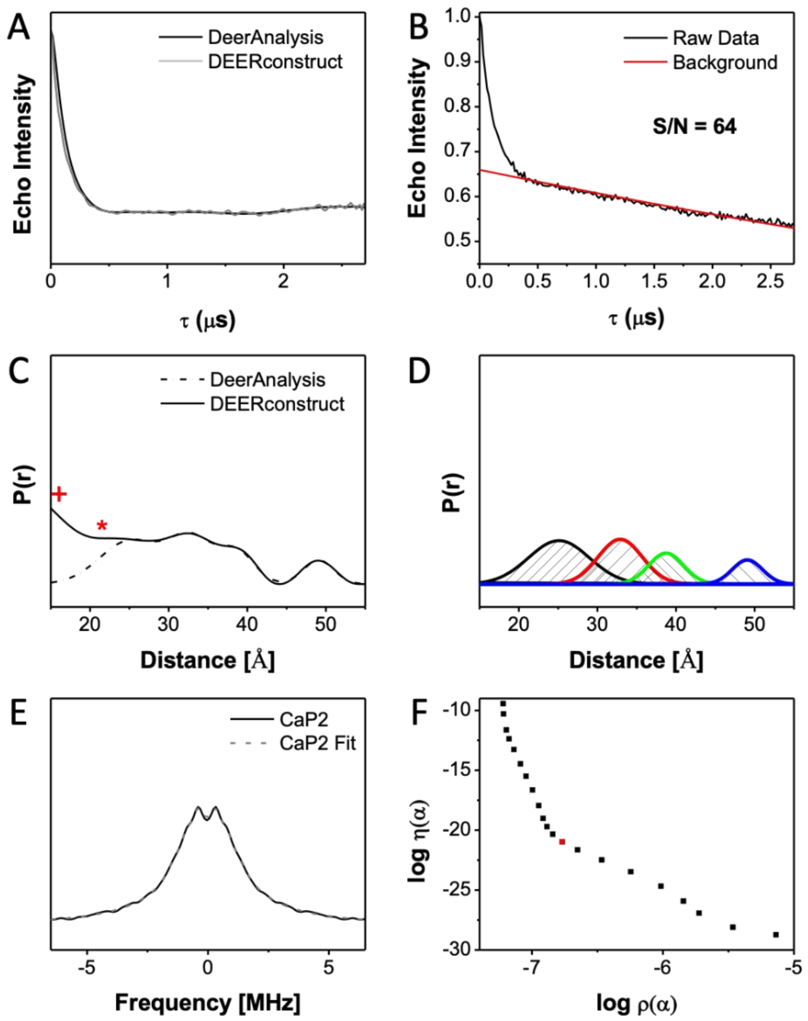

Figure S16. DEER data for CaP2-bound HIV-1 PR DRV4, pH 5.0 **A**) Background corrected dipolar evolution curve after the long pass filter in DeerAnalysis (black) and the simulated curve from DEERconstruct (gray); **B**) Raw dipolar evolution curve and background, the signal to noise ratio (S/N) is shown inset, where the signal is the DEER modulation depth and the noise is 2 times of the standard deviation of the noise curve; **C**) The corresponding distance profile generated via TKR analysis by DeerAnalysis (black) and the theoretical curve generated from the Gaussian reconstruction by DEERconstruct (gray), asterisks indicate that peaks are within the suppression range, "+" indicates that the peak is presumed to be an artifact of processing as it is near the lower limit of the generally accepted range that is measurable using DEER; **D**) The individual Gaussian functions used in the reconstruction; **E**) Frequency domain spectrum; **F**) L-curve derived from TKR fit to obtain the optimal regulation parameter, the optimal regulation parameter is plot in red.

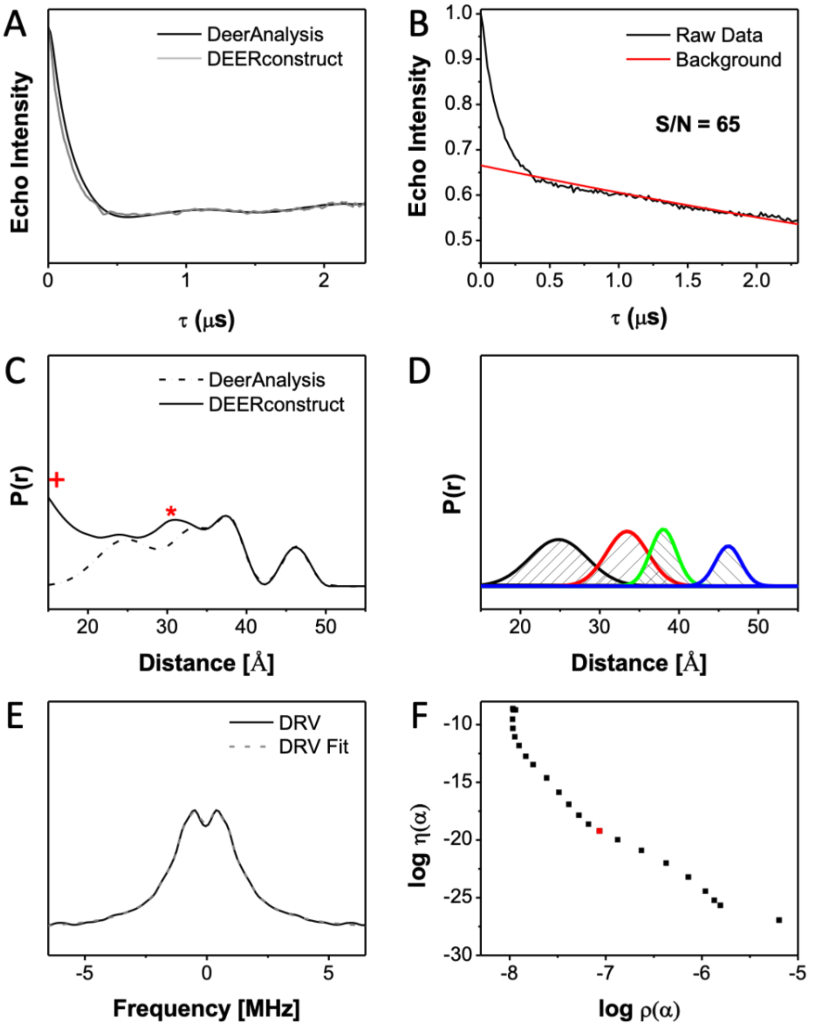

Figure S17. DEER data for DRV-bound HIV-1 PR DRV4, pH 5.0 **A**) Background corrected dipolar evolution curve after the long pass filter in DeerAnalysis (black) and the simulated curve from DEERconstruct (gray); **B**) Raw dipolar evolution curve and background, the signal to noise ratio (S/N) is shown inset, where the signal is the DEER modulation depth and the noise is 2 times of the standard deviation of the noise curve; **C**) The corresponding distance profile generated via TKR analysis by DeerAnalysis (black) and the theoretical curve generated from the Gaussian reconstruction by DEERconstruct (gray), asterisks indicate that peaks are within the suppression range, “+” indicates that the peak is presumed to be an artifact of processing as it is near the lower limit of the generally accepted range that is measurable using DEER; **D**) The individual Gaussian functions used in the reconstruction; **E**) Frequency domain spectrum; **F**) L-curve derived from TKR fit to obtain the optimal regulation parameter, the optimal regulation parameter is plot in red.

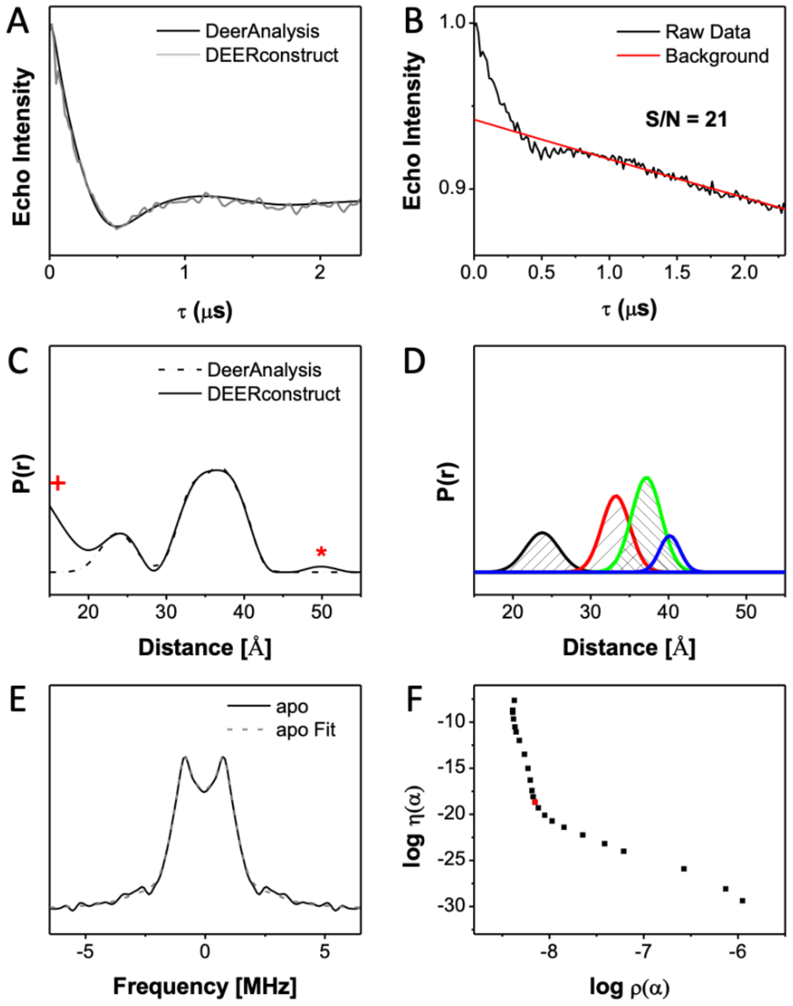

Figure S18. DEER data for apo HIV-1 PR DRV5, pH 5.0 **A**) Background corrected dipolar evolution curve after the long pass filter in DeerAnalysis (black) and the simulated curve from DEERconstruct (gray); **B**) Raw dipolar evolution curve and background, the signal to noise ratio (S/N) is shown inset, where the signal is the DEER modulation depth and the noise is 2 times of the standard deviation of the noise curve; **C**) The corresponding distance profile generated via TKR analysis by DeerAnalysis (black) and the theoretical curve generated from the Gaussian reconstruction by DEERconstruct (gray), asterisks indicate that peaks are within the suppression range, "+" indicates that the peak is presumed to be an artifact of processing as it is near the lower limit of the generally accepted range that is measurable using DEER; D) The individual Gaussian functions used in the reconstruction; **E**) Frequency domain spectrum; **F**) L-curve derived from TKR fit to obtain the optimal regulation parameter, the optimal regulation parameter is plot in red.

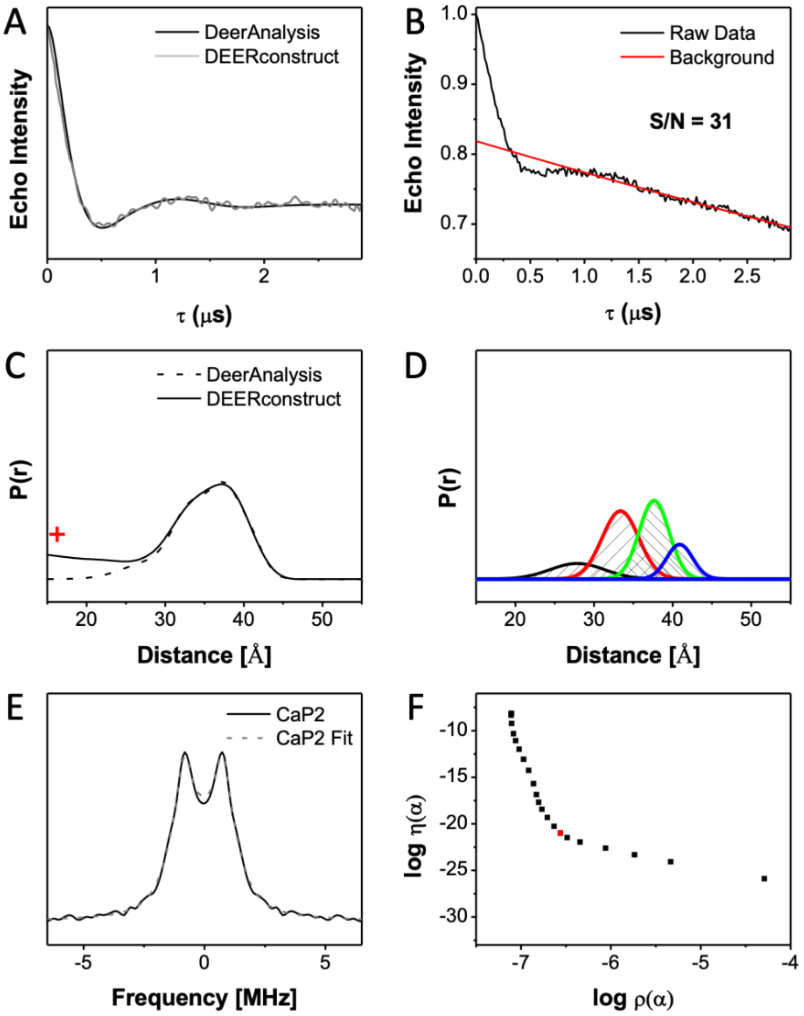

Figure S19. DEER data for CaP2-bound HIV-1 PR DRV5, pH 5.0 **A**) Background corrected dipolar evolution curve after the long pass filter in DeerAnalysis (black) and the simulated curve from DEERconstruct (gray); **B**) Raw dipolar evolution curve and background, the signal to noise ratio (S/N) is shown inset, where the signal is the DEER modulation depth and the noise is 2 times of the standard deviation of the noise curve; **C**) The corresponding distance profile generated via TKR analysis by DeerAnalysis (black) and the theoretical curve generated from the Gaussian reconstruction by DEERconstruct (gray), "+" indicates that the peak is presumed to be an artifact of processing as it is near the lower limit of the generally accepted range that is measurable using DEER; **D**) The individual Gaussian functions used in the reconstruction; **E**) Frequency domain spectrum; **F)** L-curve derived from TKR fit to obtain the optimal regulation parameter, the optimal regulation parameter is plot in red.

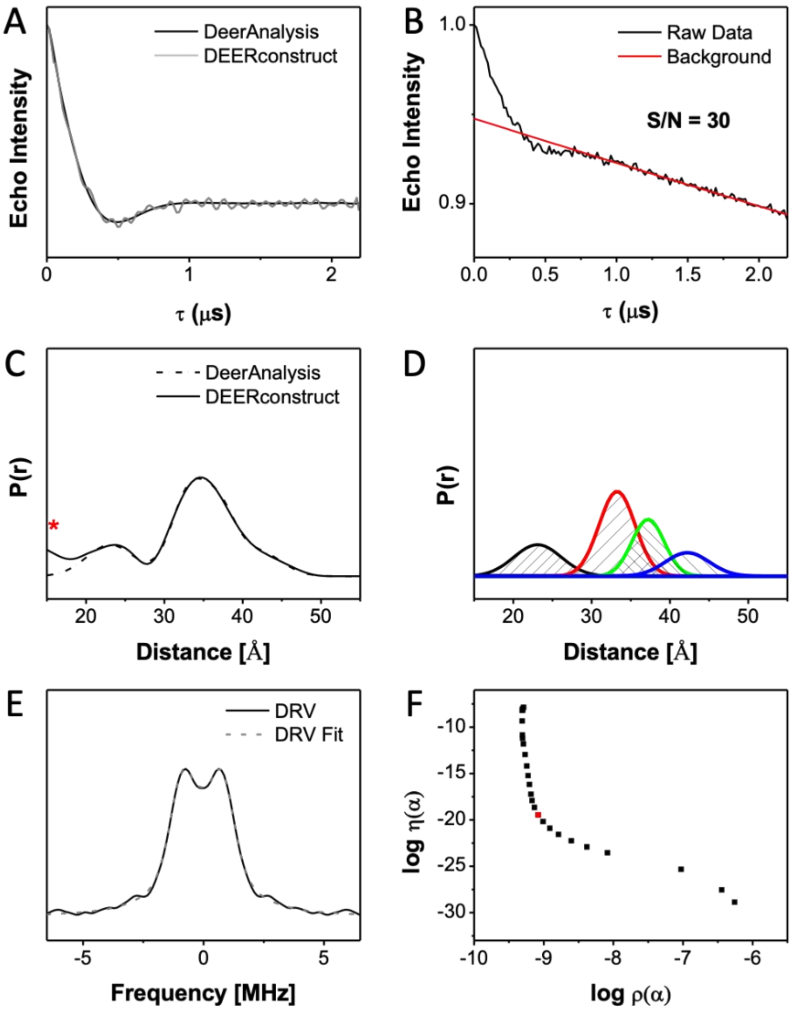

Figure S20. DEER data for DRV-bound HIV-1 PR DRV5, pH 5.0 **A**) Background corrected dipolar evolution curve after the long pass filter in DeerAnalysis (black) and the simulated curve from DEERconstruct (gray); **B**) Raw dipolar evolution curve and background, the signal to noise ratio (S/N) is shown inset, where the signal is the DEER modulation depth and the noise is 2 times of the standard deviation of the noise curve; **C**) The corresponding distance profile generated via TKR analysis by DeerAnalysis (black) and the theoretical curve generated from the Gaussian reconstruction by DEERconstruct (gray), asterisks indicate that peaks are within the suppression range; **D**) The individual Gaussian functions used in the reconstruction; **E**) Frequency domain spectrum; **F**) L-curve derived from TKR fit to obtain the optimal regulation parameter, the optimal regulation parameter is plot in red.

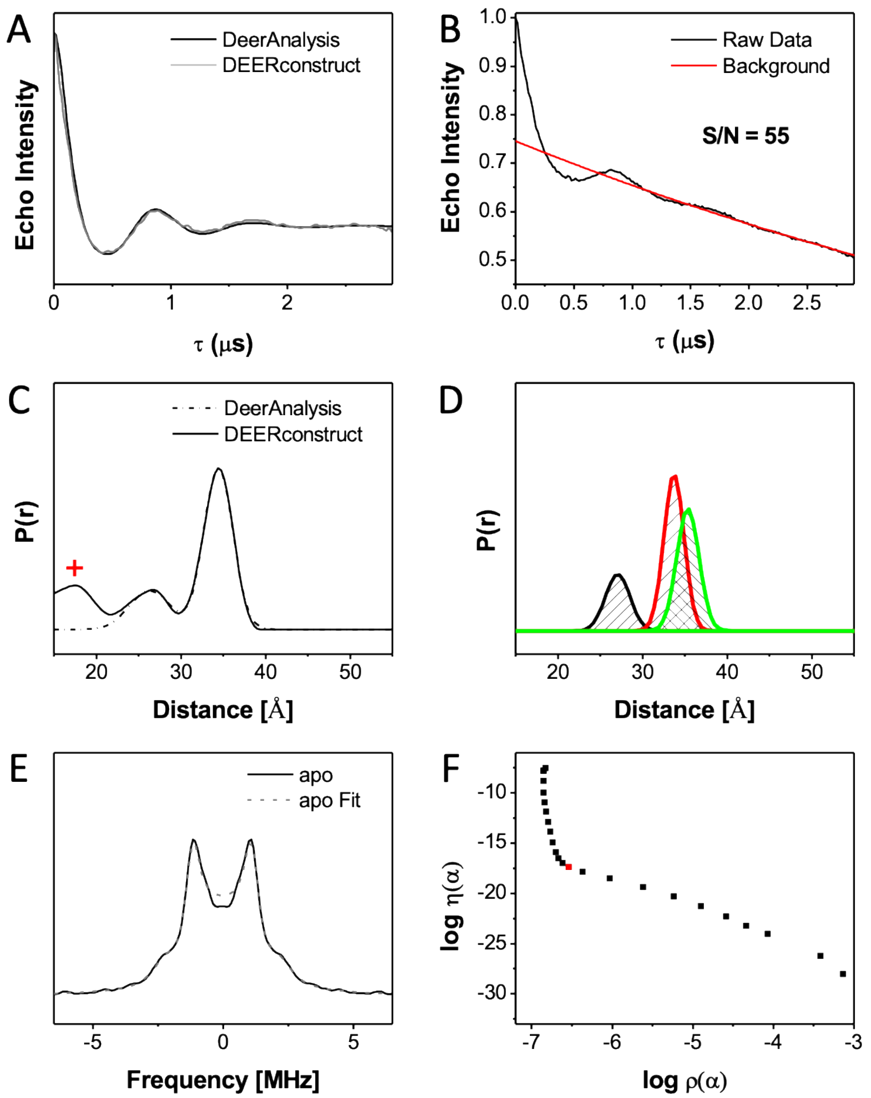

Figure S21. DEER data for apo HIV-1 PR DRV6, pH 5.0 **A**) Background corrected dipolar evolution curve after the long pass filter in DeerAnalysis (black) and the simulated curve from DEERconstruct (gray); B) Raw dipolar evolution curve and background, the signal to noise ratio (S/N) is shown inset, where the signal is the DEER modulation depth and the noise is 2 times of the standard deviation of the noise curve; **C**) The corresponding distance profile generated via TKR analysis by DeerAnalysis (black) and the theoretical curve generated from the Gaussian reconstruction by DEERconstruct (gray), "+" indicates that the peak is presumed to be an artifact of processing as it is near the lower limit of the generally accepted range that is measurable using DEER; **D**) The individual Gaussian functions used in the reconstruction; **E**) Frequency domain spectrum; **F**) L-curve derived from TKR fit to obtain the optimal regulation parameter, the optimal regulation parameter is plot in red.

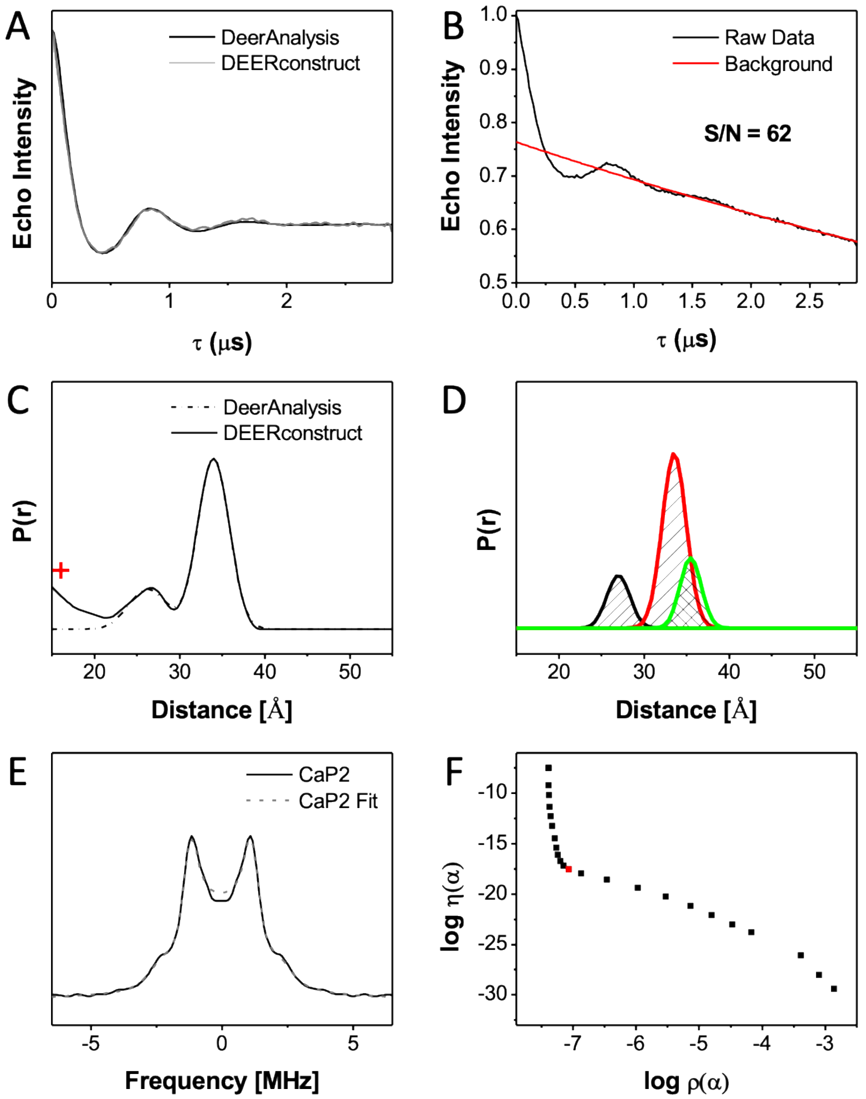

Figure S22. DEER data for CaP2-bound HIV-1 PR DRV6, pH 5.0 **A**) Background corrected dipolar evolution curve after the long pass filter in DeerAnalysis (black) and the simulated curve from DEERconstruct (gray); **B**) Raw dipolar evolution curve and background, the signal to noise ratio (S/N) is shown inset, where the signal is the DEER modulation depth and the noise is 2 times of the standard deviation of the noise curve; **C**) The corresponding distance profile generated via TKR analysis by DeerAnalysis (black) and the theoretical curve generated from the Gaussian reconstruction by DEERconstruct (gray), "+" indicates that the peak is presumed to be an artifact of processing as it is near the lower limit of the generally accepted range that is measurable using DEER; **D**) The individual Gaussian functions used in the reconstruction; **E**) Frequency domain spectrum; **F**) L-curve derived from TKR fit to obtain the optimal regulation parameter, the optimal regulation parameter is plot in red.

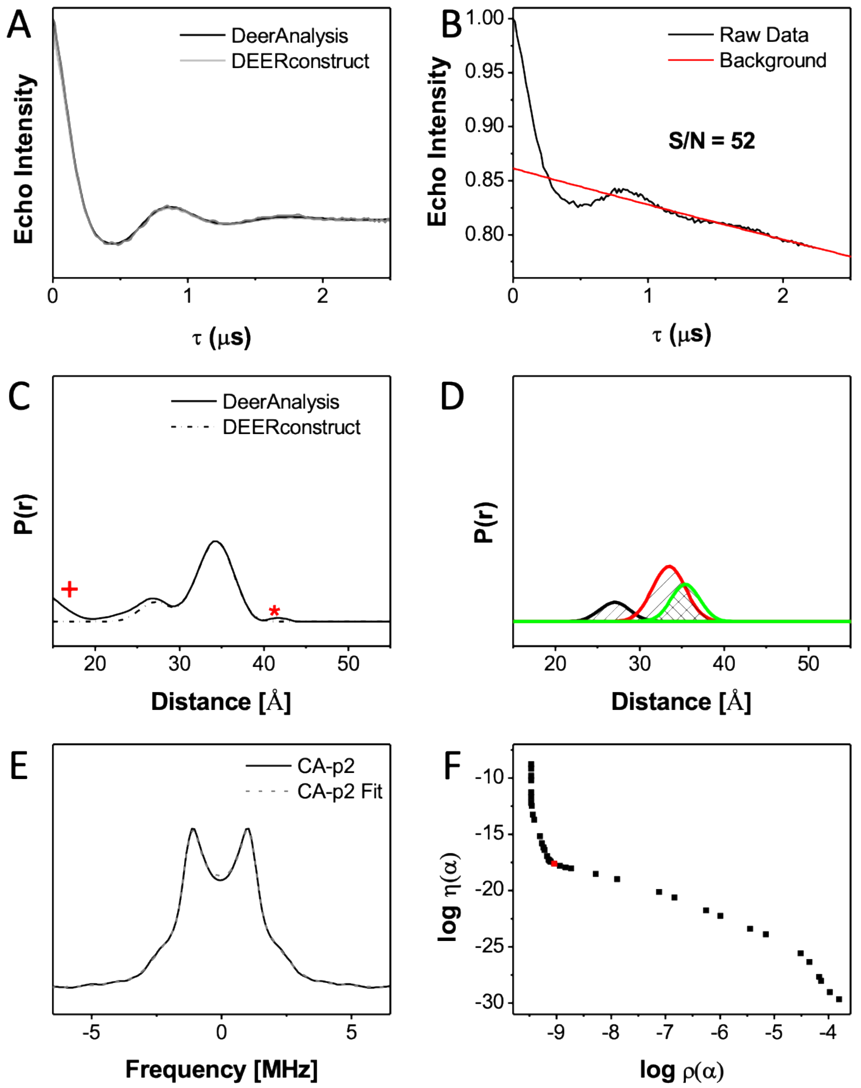

Figure S23. DEER data for DRV-bound HIV-1 PR DRV6, pH 5.0 **A**) Background corrected dipolar evolution curve after the long pass filter in DeerAnalysis (black) and the simulated curve from DEERconstruct (gray); **B**) Raw dipolar evolution curve and background, the signal to noise ratio (S/N) is shown inset, where the signal is the DEER modulation depth and the noise is 2 times of the standard deviation of the noise curve; **C**) The corresponding distance profile generated via TKR analysis by DeerAnalysis (black) and the theoretical curve generated from the Gaussian reconstruction by DEERconstruct (gray), asterisks indicate that peaks are within the suppression range, "+" indicates that the peak is presumed to be an artifact of processing as it is near the lower limit of the generally accepted range that is measurable using DEER; **D**) The individual Gaussian functions used in the reconstruction; **E**) Frequency domain spectrum; **F**) L-curve derived from TKR fit to obtain the optimal regulation parameter, the optimal regulation parameter is plot in red.

Table S2. DEER population analysis via Gaussian reconstruction.

	Curled (Å)				Closed (Å)				Semi-open (Å)				Wide-open (Å)			
**Constructs**	**Peak position**	**Peak Error**	**FWHM**	**FWHM Error**	**Peak position**	**Peak Error**	**FWHM**	**FWHM Error**	**Peak position**	**Peak Error**	**FWHM**	**FWHM Error**	**Peak position**	**Peak Error**	**FWHM**	**FWHM Error**
**DRV1 unbound**	26.7	0.7	8.0	2.1	32.4	1.5	5.7	1.3	37.4	3.1	5.0	2.7	40.9	0.6	5.2	0.7
**DRV1 with CaP2**	26.4	1.0	9.9	3.8	32.7	0.3	7.7	1.6	37.7	0.4	8.1	2.5	43.8	0.3	8.0	0.8
**DRV1 with DRV**	27.0	0.8	8.2	3.2	32.8	0.1	6.8	0.4	38.2	0.5	7.2	0.8	42.8	0.5	6.2	1.0
**DRV2 unbound**	28.8	1.9	6.3	2.2	33.4	2.4	5.6	0.3	38.2	0.4	5.7	2.1	41.5	0.3	4.9	0.4
**DRV2 with CaP2**	25.2	1.4	10.2	3.6	32.3	0.3	7.6	1.1	38.1	0.6	6.1	1.0	41.3	0.3	5.3	0.4
**DRV2 with DRV**	27.4	1.8	7.5	2.6	32.8	1.3	6.4	1.2	37.9	0.5	6.0	0.6	41.5	0.2	5.3	0.3
**DRV3 unbound**	24.2	0.5	9.1	0.9	32.1	0.3	5.9	1.4	36.3	0.7	4.9	0.7	44.4	0.1	5.9	0.2
**DRV3 unbound @pH3**	23.1	0.2	11.5	3.1	32.4	0.0	4.6	0.1	37.0	0.1	3.1	0.9	40.8	0.7	5.7	2.8
**DRV3 with CaP2**	23.2	0.7	5.0	1.7	32.6	0.0	4.8	0.1	36.2	0.5	6.0	0.9	48.6	0.0	5.8	0.2
**DRV3 with DRV**					32.7	0.1	4.2	0.1								
**DRV4 unbound**	26.3	0.9	10.3	3.1	32.8	0.6	8.0	1.2	38.9	0.4	5.4	0.6	48.4	0.0	4.8	0.0
**DRV4 with CaP2**	25.0	1.3	10.3	1.4	32.8	0.2	8.3	1.6	38.8	0.4	5.9	0.7	49.1	0.0	5.3	0.0
**DRV4 with DRV**	24.4	1.1	10.2	1.3	33.0	1.3	7.1	0.7	37.9	0.4	4.9	0.8	46.2	0.0	4.5	0.0
**DRV5 unbound**	24.1	0.8	5.4	1.6	33.1	0.3	4.8	0.6	37.0	0.3	5.8	1.4	39.8	0.9	3.7	0.3
**DRV5 with CaP2**	27.7	0.9	9.8	2.3	33.2	0.5	6.5	0.1	37.6	0.3	5.8	1.4	40.9	0.5	4.8	0.7
**DRV5 with DRV**	23.2	0.3	8.0	1.0	33.2	0.1	6.4	0.2	37.2	0.2	6.0	1.0	42.5	0.9	7.6	0.5
**DRV6 unbound**	26.4	0.1	6.8	0.1	33.6	0.3	3.9	0.6	35.5	0.1	3.5	0.2				
**DRV6 with CaP2**	26.3	0.1	6.7	1.3	33.5	0.2	4.4	1.0	35.4	0.8	3.8	1.5				
**DRV6 with DRV**	26.8	0.6	6.3	3.8	33.5	0.1	5.0	1.2	35.6	0.6	4.5	0.8				

The data analysis proceeds first by TKR analysis of the DEER echo curve to give a distance profile. DEERAnalysis2019 provides an estimate of error based upon choosing an optimal regularization parameter from an L-curve (panel F in Figs SI-5-23). This profile is then fit to a linear combination of Gaussian functions using DEERconstruct (Casey et al. **2015** *Methods in Enzymology*). When using DEERconstruct, the user chooses initial parameters for peak positions. Typically, these parameters are free to vary, yet we choose initial parameters based upon our model where the semi-open distance of ~36 Å was determined from modelling of X-ray structures, MD simulations and original data on subtype B and with the closed distance determined to be 33Å analogously. Wide open and curled tucked distances come from MD simulations and EPR data. Our software allows for peak picking based upon the maximum value seen. Clearly in cases where there is a broad distribution there is more error or ambiguity. In those cases, 33Å and 36 Å are set as the initial values and allowed to vary only slightly (0.5Å and 1 Å, respectively) based upon the structural model from X-ray data. We typically also restrict the breadth of the "closed" state; as we have "control" data for many non-drug resistant constructs that show a rather narrow ranging from 4 - 6 Å for FWHM. A broad distribution in width likely indicate heterogeneity of that conformational state. Error reported here is representative from 3x STD from three separate fitting approaches with DEERconstruct for a given regularization parameter, where the initial parameter values were altered. The closed conformation is the best defined as we have numerous data sets where this conformation is obtained from protease with inhibitor.

6. Population Analysis Significance Z-Test

Table S3. Relative Populations of Conformational States Determined From DEER Analysis.

	Closed	Curled	Wide-Open	Semi-open	wide-open + curled	difference in semi-open to B
B	3 ± 4	0 ± 4	7 ± 4	90 ± 4	7 ± 6	0 ± 6
DRV1	21 ± 5	31 ± 5	35 ± 5	13 ± 5	66 ± 7	-77 ± 6
DRV2	21 ± 5	15 ± 5	27 ± 5	37 ± 5	42 ± 7	-53 ± 6
DRV3	61 ± 5	27 ± 5	7 ± 5	5 ± 5	34 ± 7	-85 ± 6
DRV4	26 ± 5	37 ± 5	15 ± 5	22 ± 5	52 ± 7	-68 ± 6
DRV5	30 ± 5	19 ± 5	11 ± 5	40 ± 5	30 ± 7	-50 ± 6
DRV6	44 ± 5	19 ± 5	0 ± 5	37 ± 5	19 ± 7	-53 ± 6

Table S4. Z-test for Evaluating the Difference in the Semi-open Population Relative to Subtype B.

	Semi-Open Difference compared to Subtype B	STD of error	Z score	Probability (p) at calculated Z score
B	0			
DRV1	53	6.40	12	2.626 x 10^-23
DRV2	85	6.40	8.3	1.263 x 10^-16
DRV3	68	6.40	13	3.276 x 10^-40
DRV4	50	6.40	10	2.432 x 10^-26
DRV5	53	6.40	7.8	5.81 x 10^-15
DRV6	77	6.40	8.3	1.263 x 10^-16

1. The difference between distance measurements is assumed have a normal distribution
2. The mean for the normal distribution is zero (why we have "-0" in the formula to calculate Z score)
3. Probability (p) at calculated Z score: Go to "wolframalpha.com" and type in "12.025 standard deviation"
4. A two-tailed test was utilized.
5. All are greater than 99.99% statistically significantly different than B.

Table S5. Z-test for Evaluating the Difference in the Open-Like = Wide-Open+ Curled Populations Relative to Subtype B.

	Semi-Open Difference compared to Subtype B	STD of error	Z score	Probability (p) at calculated Z score
B	0			
DRV1	59	9.06	6.5	7.269 x 10^-11
DRV2	35	9.06	3.9	1.111 x 10^-4
DRV3	27	9.06	3.0	0.002873
DRV4	45	9.06	5.0	6.73 x 10^-7
DRV5	23	9.06	2.5	0.01112
DRV6	12	9.06	1.32	0.1852

1. The difference between distance measurements is assumed have a normal distribution
2. The mean for the normal distribution is zero (why we have "-0" in the formula to calculate Z score)
3. Probability (p) at calculated Z score: Go to "wolframalpha.com" and type in "12.025 standard deviation"
4. A two-tailed test was utilized
5. All except DRV6 are greater than 98% statistically significant.
6. DRV6 is only 87% significantly different.

Table S6. Z-test for Evaluating the Difference in the Closed Populations Relative to Subtype B.

	**Semi-Open Difference compared to Subtype B**	**STD of error**	**Z score**	**Probability (p) at calculated Z score**
B	0			
DRV1	18	7.55	2.4	0.01112
DRV2	18	7.55	2.4	0.01112
DRV3	58	7.55	7.7	5.81 x 10^-15
DRV4	23	7.55	3.0	1.111 x 10^-4
DRV5	27	7.55	3.6	1.111 x 10^-4
DRV6	41	7.55	5.4	6.73 x 10^-7

1. The difference between distance measurements is assumed have a normal distribution
2. The mean for the normal distribution is zero (why we have "-0" in the formula to calculate Z score)
3. Probability (p) at calculated Z score: Go to "wolframalpha.com" and type in "12.025 standard deviation"
4. A two-tailed test was utilized
5. All are greater than 98% statistically significant.

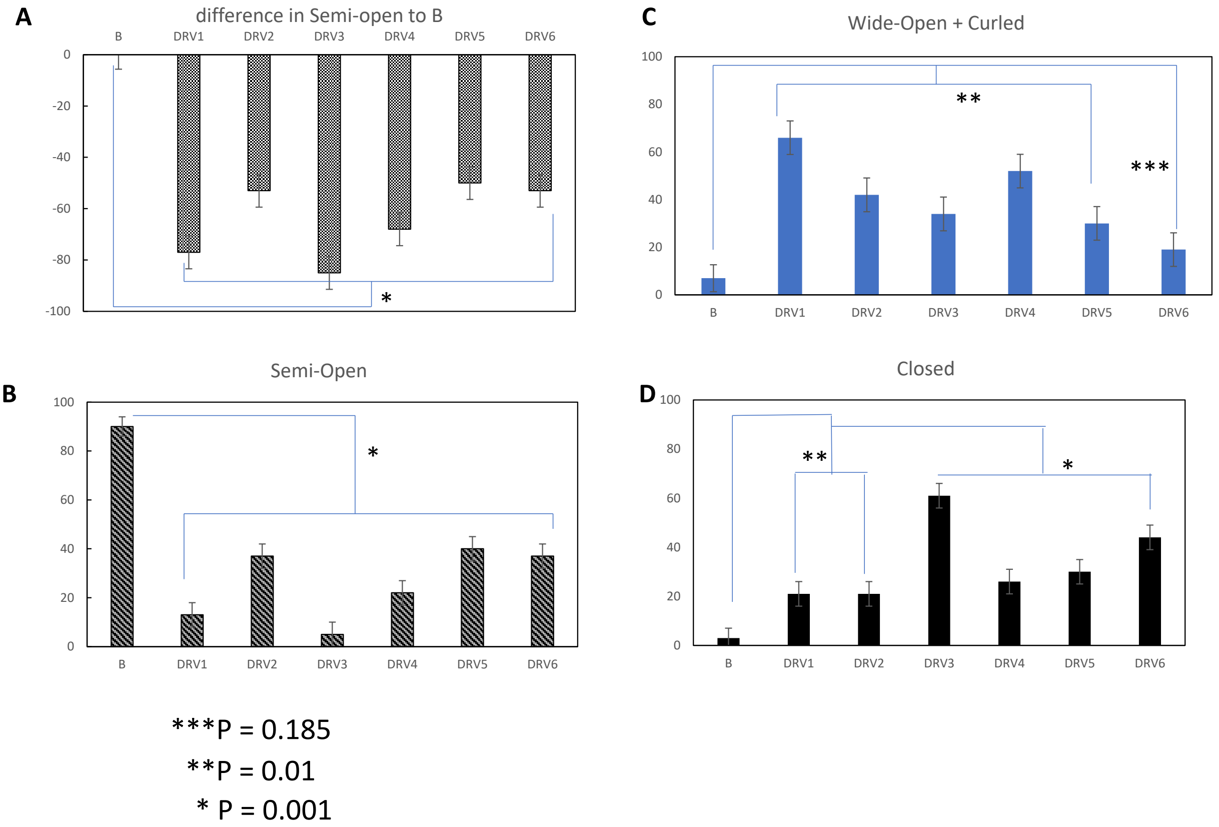

Figure S24. Analysis of significance of population differences for conformational sampling of DRV constructs relative to Subtype B.

References:

1. Kear, J. L., Galiano, L., Veloro, A. M., Harris, J., Busenlehner, L. S., and Fanucci, G. E. (2011) Monitoring the autoproteolysis of HIV-1 Protease by Site-Directed Spin-Labeling and Electron Paramagnetic Resonance Spectroscopy, *J. Biophys. Chem. 2*, 137-146.
2. Blackburn, M. E., Veloro, A. M., and Fanucci, G. E. (2009) Monitoring inhibitor-induced conformational population shifts in HIV-1 protease by pulsed EPR spectroscopy, *Biochemistry 48*, 8765-8767.

3. de Vera, I. M., Smith, A. N., Dancel, M. C., Huang, X., Dunn, B. M., and Fanucci, G. E. (2013) Elucidating a relationship between conformational sampling and drug resistance in HIV-1 protease, *Biochemistry 52*, 3278-3288.
4. Jeschke, G., Chechik, V., Ionita, P., Godt, A., Zimmermann, H., Banham, J., Timmel, C. R., Hilger, D., and Jung, H. (2006) DeerAnalysis2006 - A Comprehensive Software Package for Analyzing Pulsed ELDOR Data, *Appl. Mag. Reson. 30*, 473-498.
5. Casey, T. M., and Fanucci, G. E. (2015) Spin labeling and Double Electron-Electron Resonance (DEER) to Deconstruct Conformational Ensembles of HIV Protease, *Methods Enzymol 564*, 153-187.
6. de Vera, I. M., Blackburn, M. E., and Fanucci, G. E. (2012) Correlating conformational shift induction with altered inhibitor potency in a multidrug resistant HIV-1 protease variant, *Biochemistry 51*, 7813-7815.
7. De Vera, I. M., Blackburn, M. E., Galiano, L., and Fanucci, E. (2013) Pulsed EPR distance measurements in soluble proteins by site-directed spin labeling (SDSL), *Curr Prot Protein Sci 74*, 17.17.
8. Huang, X., de Vera, I. M., Veloro, A. M., Blackburn, M. E., Kear, J. L., Carter, J. D., Rocca, J. R., Simmerling, C., Dunn, B. M., and Fanucci, G. E. (2012) Inhibitor-induced conformational shifts and ligand-exchange dynamics for HIV-1 protease measured by pulsed EPR and NMR spectroscopy, *J Phys Chem B 116*, 14235-14244.
9. Huang, X., Britto, M. D., Kear-Scott, J. L., Boone, C. D., Rocca, J. R., Simmerling, C., McKenna, R., Bieri, M., Gooley, P. R., Dunn, B. M., and Fanucci, G. E. (2014) The role of select subtype polymorphisms on HIV-1 protease conformational sampling and dynamics, *J Biol Chem 289*, 17203-17214.
10. Liu, H. L., Huang, X., Hu, L., Pham, L., Poole, K. M., Tang, Y., Mahon, B. P., Tang, W., Li, K., Goldfarb, N. E., Dunn, B. M., McKenna, R., and Fanucci, G. E. (2016) Molecular Insights into the Effects of Natural Polymorphisms in the Hinge Region of HIV-1 Protease on Protein Structure, Dynamics and Evolution, *J Biol Chem 291, 22741-22756*.
